# A novel common variant in *DCST2* is associated with length in early life and height in adulthood

**DOI:** 10.1093/hmg/ddu510

**Published:** 2014-10-03

**Authors:** Ralf J.P. van der Valk, Eskil Kreiner-Møller, Marjolein N. Kooijman, Mònica Guxens, Evangelia Stergiakouli, Annika Sääf, Jonathan P. Bradfield, Frank Geller, M. Geoffrey Hayes, Diana L. Cousminer, Antje Körner, Elisabeth Thiering, John A. Curtin, Ronny Myhre, Ville Huikari, Raimo Joro, Marjan Kerkhof, Nicole M. Warrington, Niina Pitkänen, Ioanna Ntalla, Momoko Horikoshi, Riitta Veijola, Rachel M. Freathy, Yik-Ying Teo, Sheila J. Barton, David M. Evans, John P. Kemp, Beate St Pourcain, Susan M. Ring, George Davey Smith, Anna Bergström, Inger Kull, Hakon Hakonarson, Frank D. Mentch, Hans Bisgaard, Bo Chawes, Jakob Stokholm, Johannes Waage, Patrick Eriksen, Astrid Sevelsted, Mads Melbye, Early Genetics and Lifecourse Epidemiology (EAGLE) Consortium, Cornelia M. van Duijn, Carolina Medina-Gomez, Albert Hofman, Johan C. de Jongste, H. Rob Taal, André G. Uitterlinden, Genetic Investigation of ANthropometric Traits (GIANT) Consortium, Loren L. Armstrong, Johan Eriksson, Aarno Palotie, Mariona Bustamante, Xavier Estivill, Juan R. Gonzalez, Sabrina Llop, Wieland Kiess, Anubha Mahajan, Claudia Flexeder, Carla M.T. Tiesler, Clare S. Murray, Angela Simpson, Per Magnus, Verena Sengpiel, Anna-Liisa Hartikainen, Sirkka Keinanen-Kiukaanniemi, Alexandra Lewin, Alexessander Da Silva Couto Alves, Alexandra I. Blakemore, Jessica L. Buxton, Marika Kaakinen, Alina Rodriguez, Sylvain Sebert, Marja Vaarasmaki, Timo Lakka, Virpi Lindi, Ulrike Gehring, Dirkje S. Postma, Wei Ang, John P. Newnham, Leo-Pekka Lyytikäinen, Katja Pahkala, Olli T. Raitakari, Kalliope Panoutsopoulou, Eleftheria Zeggini, Dorret I. Boomsma, Maria Groen-Blokhuis, Jorma Ilonen, Lude Franke, Joel N. Hirschhorn, Tune H. Pers, Liming Liang, Jinyan Huang, Berthold Hocher, Mikael Knip, Seang-Mei Saw, John W. Holloway, Erik Melén, Struan F.A. Grant, Bjarke Feenstra, William L. Lowe, Elisabeth Widén, Elena Sergeyev, Harald Grallert, Adnan Custovic, Bo Jacobsson, Marjo-Riitta Jarvelin, Mustafa Atalay, Gerard H. Koppelman, Craig E. Pennell, Harri Niinikoski, George V. Dedoussis, Mark I. Mccarthy, Timothy M. Frayling, Jordi Sunyer, Nicholas J. Timpson, Fernando Rivadeneira, Klaus Bønnelykke, Vincent W.V. Jaddoe

**Affiliations:** 1 Department of Epidemiology,; 2 Department of Paediatrics,; 3 The Generation R Study Group,; 4 Department of Internal Medicine, Erasmus Medical Center, Rotterdam, The Netherlands,; 5 Copenhagen Prospective Studies on Asthma in Childhood, Faculty of Health Sciences, University of Copenhagen & Danish Pediatric Asthma Center, Copenhagen University Hospital, Gentofte, Denmark,; 6 Centre for Research in Environmental Epidemiology (CREAL), Barcelona, Spain,; 7 CIBER Epidemiología y Salud Pública (CIBERESP), Spain,; 8 Pompeu Fabra University (UPF), Barcelona, Catalonia, Spain,; 9 MRC Integrative Epidemiology Unit ,; 10 Avon Longitudinal Study of Parents and Children (ALSPAC), School of Social and Community Medicine,; 11 School of Oral and Dental Sciences, University of Bristol, Bristol, UK,; 12 Institute of Environmental Medicine, Karolinska Institutet, Stockholm, Sweden,; 13 Center for Applied Genomics, Abramson Research Center,; 14 Division of Human Genetics, The Children's Hospital of Philadelphia, Philadelphia, PA 19104, USA,; 15 Department of Epidemiology Research, Statens Serum Institut, Copenhagen, Denmark,; 16 Division of Endocrinology, Metabolism and Molecular Medicine,; 17 Northwestern University Feinberg School of Medicine, Chicago, IL 60611, USA,; 18 Institute for Molecular Medicine Finland,; 19 Diabetes and Obesity Research Program, University of Helsinki, Helsinki, Finland,; 20 Center of Pediatric Research, University Hospital Center Leipzig, University of Leipzig, Leipzig, Germany,; 21 Division of Metabolic and Nutritional Medicine, Dr. von Hauner Children's Hospital, University of Munich Medical Center, Munich, Germany,; 22 Institute of Epidemiology I,; 23 Institute of Epidemiology II,; 24 Research Unit for Molecular Epidemiology, Helmholtz Zentrum München – German Research Center for Environmental Health, Neuherberg, Germany,; 25 Centre for Respiratory Medicine and Allergy, Institute of Inflammation and Repair, University of Manchester and University Hospital of South Manchester, Manchester Academic Health Sciences Centre, Manchester, UK,; 26 Division Epidemiology, Department Genes and Environment,; 27 Division Epidemiology, Norwegian Institute of Public Health, Oslo, Norway,; 28 Institute of Health Sciences,; 29 Institute of Clinical Medicine/Obstetrics and Gynecology,; 30 Biocenter Oulu, University of Oulu, Oulu, Finland,; 31 Institute of Biomedicine, Physiology,; 32 Department of Clinical Microbiology, University of Eastern Finland, Kuopio, Finland,; 33 Department of Epidemiology,; 34 Groningen Research Institute for Asthma and COPD,; 35 Department of Pulmonology,; 36 Beatrix Children's Hospital, Pediatric Pulmonology and Pediatric Allergy, University of Groningen, University Medical Center Groningen, Groningen, The Netherlands,; 37 School of Women's and Infants’ Health, The University of Western Australia, Perth, Australia,; 38 University of Queensland Diamantina Institute, Translational Research Institute, Brisbane, Queensland, Australia,; 39 Research Centre of Applied and Preventive Cardiovascular Medicine,; 40 Immunogenetics Laboratory, University of Turku, Turku, Finland,; 41 Department of Health Sciences, University of Leicester, Leicester LE1 7RH, UK,; 42 Department of Nutrition and Dietetics, Harokopio University of Athens, Athens 11527, Greece,; 43 Wellcome Trust Centre for Human Genetics, University of Oxford, Oxford OX3 7BN, UK,; 44 Oxford Centre for Diabetes, Endocrinology and Metabolism, University of Oxford, Churchill Hospital, Oxford OX3 7LJ, UK,; 45 Department of Pediatrics, Medical Research Center,; 46 Department of Obstetrics and Gynecology and MRC Oulu, Oulu University Hospital and University of Oulu, Oulu, Finland,; 47 University of Exeter Medical School, Royal Devon and Exeter Hospital, Barrack Road, Exeter EX2 5DW, UK,; 48 Saw Swee Hock School of Public Health,; 49 Life Science Institute, National University of Singapore, Singapore,; 50 Genome Institute of Singapore, Agency for Science, Technology and Research,; 51 MRC Lifecourse Epidemiology Unit,; 52 Human Genetics and Genomic Medicine, Human Development & Health, Faculty of Medicine, University of Southampton, UK,; 53 Department of Clinical Science and Education, Södersjukhuset, Stockholm, Sweden,; 54 Sachs’ Children's Hospital, Stockholm, Sweden,; 55 Department of Pediatrics, Perelman School of Medicine, University of Pennsylvania, Philadelphia, PA, USA,; 56 Department of Medicine, Stanford School of Medicine, Stanford, USA,; 57 Analytic and Translational Genetics Unit, Department of Medicine,; 58 Psychiatric & Neurodevelopmental Genetics Unit, Department of Psychiatry, Massachusetts General Hospital, Boston, MA, USA,; 59 Program in Medical and Population Genetics,; 60 Medical and Population Genetics Program, Broad Institute of MIT and Harvard, Cambridge, MA, USA,; 61 Centre for Genomic Regulation (CRG), Barcelona, Spain,; 62 IMIM (Hospital del Mar Medical Research Institute), Barcelona, Spain,; 63 Foundation for the Promotion of Health and Biomedical Research in the Valencian Region, FISABIO-Public Health, Valencia, Spain,; 64 Department Obstetrics and Gynecology, Sahlgrenska Academy, Sahlgrenska University Hospital, Gothenburg, Sweden,; 65 Department of Epidemiology and Biostatistics, School of Public Health, Imperial College London, MRC Health Protection Agency (HPE) Centre for Environment and Health,; 66 Section of Investigative Medicine, Division of Diabetes, Endocrinology, and Metabolism, Faculty of Medicine, Imperial College, London W12 0NN, UK,; 67 Department of Psychology, Mid Sweden University, Östersund, Sweden,; 68 Kuopio Research Institute of Exercise Medicine, Kuopio, Finland,; 69 Department of Clinical Physiology and Nuclear Medicine, Kuopio University Hospital, Kuopio, Finland,; 70 Institute for Risk Assessment Sciences, Utrecht University, Utrecht, The Netherlands,; 71 Department of Clinical Chemistry, Fimlab Laboratories, Tampere, Finland,; 72 Department of Clinical Chemistry, University of Tampere School of Medicine, Tampere, Finland,; 73 Sports and Exercise Medicine Unit, Department of Physical Activity and Health, Paavo Nurmi Centre, Turku, Finland,; 74 Department of Clinical Physiology and Nuclear Medicine,; 75 Department of Pediatrics, Turku University Hospital, Turku, Finland,; 76 Wellcome Trust Sanger Institute, The Morgan Building, Wellcome Trust Genome Campus, Hinxton, Cambridgeshire CB10 1HH, UK,; 77 Department of Biological Psychology, VU University, Amsterdam, The Netherlands,; 78 EMGO Institute for Health and Care Research, Amsterdam, The Netherlands,; 79 Neuroscience Campus Amsterdam, The Netherlands,; 80 Department of Genetics, University of Groningen, University Medical Centre Groningen, The Netherlands,; 81 Division of Endocrinology and Center for Basic and Translational Obesity Research, Boston Children's Hospital, USA,; 82 Department of Genetics, Harvard Medical School, USA,; 83 Center for Biological Sequence Analysis, Department of Systems Biology, Technical University of Denmark, Denmark,; 84 Department of Biostatistics and Epidemiology, Harvard School of Public Health, Boston, USA,; 85 Shanghai Institute of Hematology, Rui Jin Hospital Affiliated with Shanghai Jiao Tong University School of Medicine, Shanghai, China,; 86 Institute of Nutritional Science, University of Potsdam, Germany,; 87 The First Affiliated Hospital of Jinan University, Guangzhou 510630, China,; 88 Center for Cardiovascular Research/Institute of Pharmacology, Charité, Berlin, Germany,; 89 Department of Pediatrics, Tampere University Hospital, Tampere, Finland,; 90 Children's Hospital, University of Helsinki and Helsinki University Central Hospital, Helsinki, Finland,; 91 Singapore Eye Research Institute, Singapore,; 92 Duke-NUS Graduate Medical School, Singapore,; 93 Unit of Primary Care, Oulu University Hospital, Kajaanintie 50, P.O.Box 20, FI-90220, Oulu 90029 OYS, Finland,; 94 Department of Children and Young People and Families, National Institute for Health and Welfare, Aapistie 1, Box 310, Oulu FI-90101, Finland and; 95 Oxford NIHR Biomedical Research Centre, Churchill Hospital, Oxford OX3 7LJ, UK

## Abstract

Common genetic variants have been identified for adult height, but not much is known about the genetics of skeletal growth in early life. To identify common genetic variants that influence fetal skeletal growth, we meta-analyzed 22 genome-wide association studies (Stage 1; *N* = 28 459). We identified seven independent top single nucleotide polymorphisms (SNPs) (*P* < 1 × 10^−6^) for birth length, of which three were novel and four were in or near loci known to be associated with adult height (*LCORL*, *PTCH1*, *GPR126* and *HMGA2*). The three novel SNPs were followed-up in nine replication studies (Stage 2; *N* = 11 995), with rs905938 in *DC-STAMP domain containing 2* (*DCST2*) genome-wide significantly associated with birth length in a joint analysis (Stages 1 + 2; *β* = 0.046, SE = 0.008, *P* = 2.46 × 10^−8^, explained variance = 0.05%). Rs905938 was also associated with infant length (*N* = 28 228; *P* = 5.54 × 10^−4^) and adult height (*N* = 127 513; *P* = 1.45 × 10^−5^). DCST2 is a DC-STAMP-like protein family member and DC-STAMP is an osteoclast cell-fusion regulator. Polygenic scores based on 180 SNPs previously associated with human adult stature explained 0.13% of variance in birth length. The same SNPs explained 2.95% of the variance of infant length. Of the 180 known adult height loci, 11 were genome-wide significantly associated with infant length (*SF3B4*, *LCORL*, *SPAG17*, *C6orf173*, *PTCH1*, *GDF5*, *ZNFX1*, *HHIP*, *ACAN*, *HLA* locus and *HMGA2*). This study highlights that common variation in *DCST2* influences variation in early growth and adult height.

## INTRODUCTION

Fetal and infancy length growth are important measures of development in early life. Early length growth seems to be associated with height in adulthood (1). It has been shown that fetal and infant growth are independently associated with higher risks of cardiovascular disease, type 2 diabetes and many other complex diseases. Previous findings suggested genetic links between fetal growth and metabolism (2,3). However, these studies mainly focused on birth weight as early growth measure. Skeletal growth is a different measure of development in early life. Skeletal growth during fetal life and infancy is a complex trait with heritability estimates of 26–72% (4). Although correlated with each other, fetal, infant and adult skeletal growth may be influenced by different genetic factors. Many common genetic variants have been identified for adult height (5), but not much is known about the genetics of skeletal growth in early life. Although, several rare genetic defects with large effects on length at birth and during infancy have been found (6,7), common genetic variants that influence normal variation in birth and infant length have not yet been identified. Therefore, we aimed to identify common genetic variants influencing early length growth, also in perspective of their effect on adult stature.

## RESULTS

**Figure 1:**
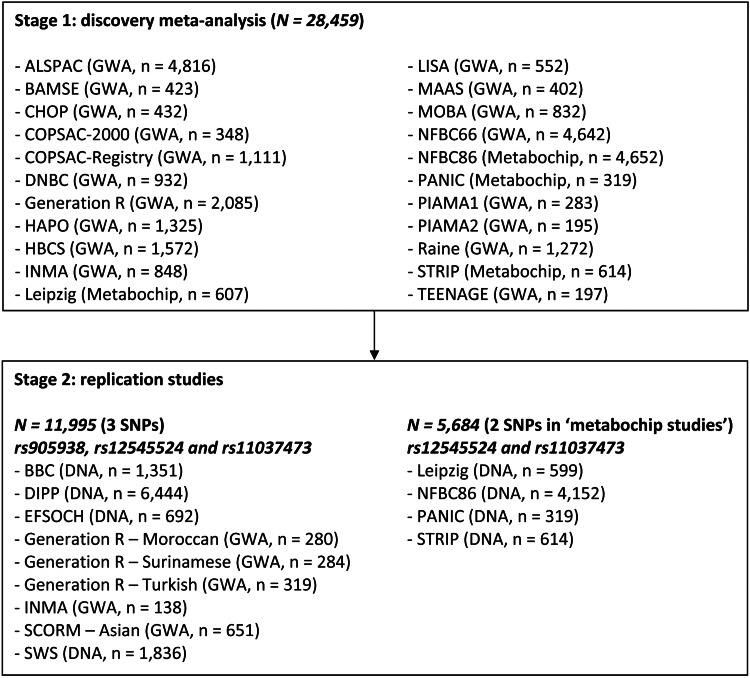
Study design.

To identify common genetic variants associated with birth length, we examined 2 201 971 million directly genotyped and imputed SNPs with birth length in 22 independent discovery studies with genome-wide association (GWA) or Metabochip data (Stage 1; *N* = 28 459; Fig. 1). Birth length was measured using standardized procedures (Supplementary Material, Tables S1 and S2). Studies with self-reported measurements were excluded a priori. Birth length was standardized using growth analyzer (http://www.growthanalyser.org), transforming birth length into sex- and age-adjusted standard deviation scores (SDS). We used the North-European 1991 reference panel to compare results between studies. We applied linear regression between number of alleles or dosages obtained from imputations and standardized birth length (full details in Materials and Methods).

### Gene identification

**Figure 2:**
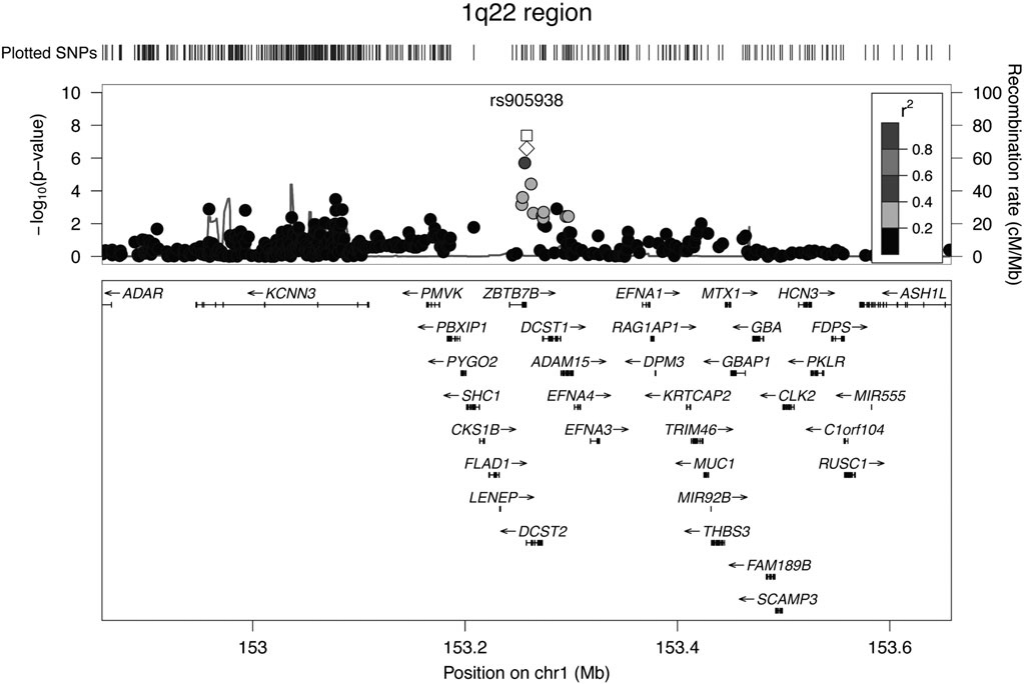
Regional association plot of 1q22 in the 22 birth length discovery studies (*N* = 28 459). SNPs are plotted with their *P* values (as −log_10_ values; left *y*-axis) as a function of genomic position (*x*-axis). Estimated recombination rates (right *y*-axis) taken from HapMap are plotted to reflect the local LD-structure around the top associated SNP (‘white open diamond’) and the correlated proxies (‘circles’ according to a black-to-gray scale from *r*^2^ = 0 to 1). The joint analysis *P* value of discovery and replication studies is reported with the ‘white square’ (*N* = 40 235).

**Figure 3:**
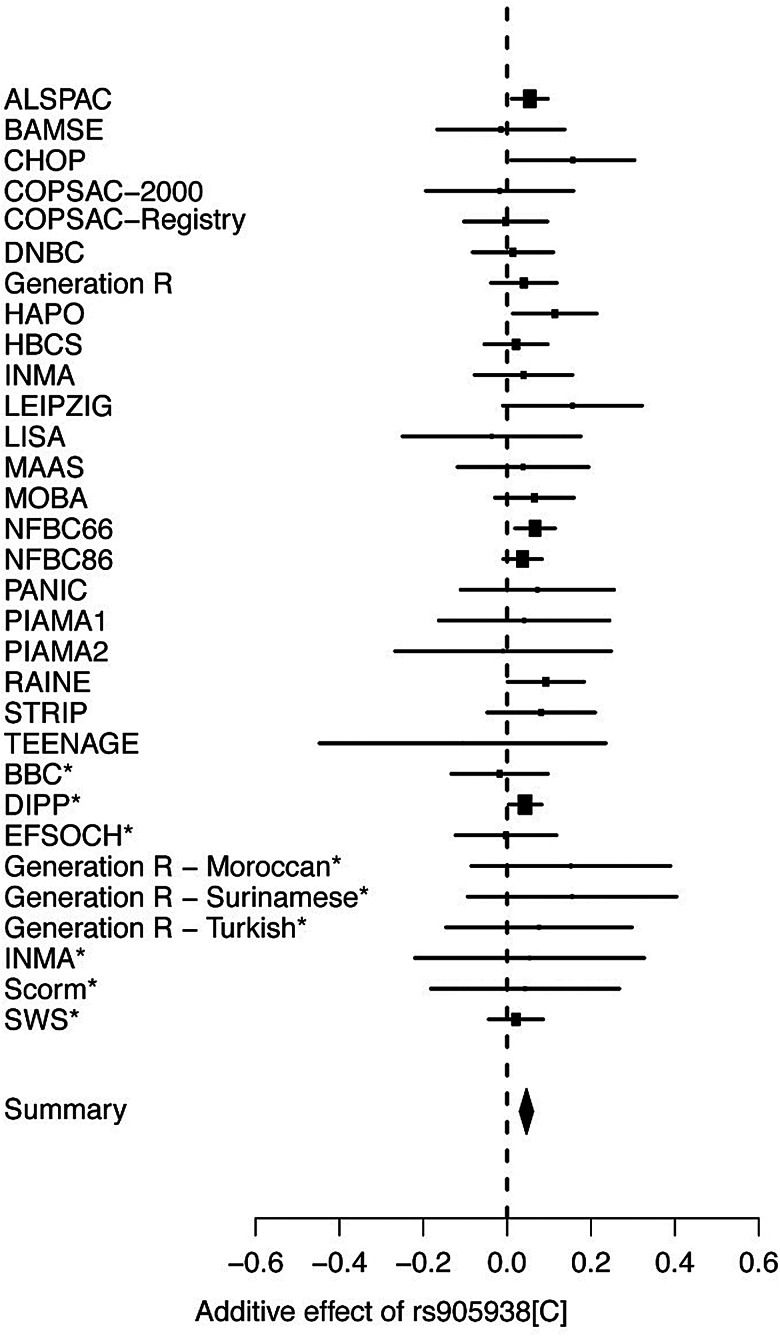
Forest plot of the associations between rs905938[C] and birth length. *Replication studies. The ‘black diamond’ indicates the overall effect size and the confidence interval of the 31 studies.

In the discovery phase (Stage 1), we found seven independent top SNPs with suggestive evidence of association (*P* < 1 × 10^−6^) with birth length (Supplementary Material, Figs. S1 and S2, *QQ*- and Manhattan plot). Four SNPs mapped to loci already known to be associated with adult height (Supplementary Material, Table S3, *LCORL*, *PTCH1*, *GPR126* and *HMGA2*) (5). The 3 SNPs reflecting potentially novel associations were taken forward in nine independent replication studies (Stage 2; *N* = 11 995; Fig. 1). Only one of the three SNPs displayed significant evidence for replication in Stage 2 and reached genome-wide significance in the joint analysis (Stages 1 + 2; *P* < 5 × 10^−8^; Table 1). This novel association arose from SNP rs905938, mapping to chromosome 1q22 in *DC-STAMP domain containing 2* (*DCST2*) (Fig. 2, regional association plot). Each C allele [minor allele frequency (MAF) = 0.24] of rs905938 was associated with an increase (standardized) of 0.046 SDS in birth length (standard error = 0.008, *P* = 2.46 × 10^−8^; explained variance = 0.05%). The genome-wide significantly associated SNP showed low degree of heterogeneity between the discovery studies (*P* = 0.93, *I*^2^ = 0%). Figure 3 shows the forest plot of the associations between rs905938[C] and birth length across all studies. Other suggestive loci in the discovery analysis are shown in Supplementary Material, Table S3 (*P* < 1 × 10^−5^). Summary statistics of all SNPs are available at http://egg-consortium.org.

**Table 1:** Summary statistics of the three novel SNPs at *P* < 1 × 10^−6^ in the discovery analysis and the replication follow-up results

Marker	MAF	*β*	SE	*P*	*n*	*I*^2^	*HetP*
Discovery (Stage 1)							
rs905938[C] at 1q22 (*DCST2*)	0.24	0.050	0.010	2.59 × 10^−7^	28 327	0.0	0.930
rs12545524[G] at 8q22.1 (near *GDF6*)	0.14	0.078	0.014	1.54 × 10^−8^	22 170	6.6	0.376
rs11037473[A] at 11p11.2 (nearest genes *TTC17*-*HSD17B12*)	0.06	−0.109	0.021	2.17 × 10^−7^	22 259	0.0	0.735
Replication (Stage 2)							
rs905938[C] at 1q22 (*DCST2*)	0.23	0.035	0.015	1.99 × 10^−2^	11 908	–	–
rs12545524[G] at 8q22.1 (near *GDF6*)	0.11	−0.012	0.017	4.67 × 10^−1^	17 614	–	–
rs11037473[A] at 11p11.2 (nearest genes *TTC17*-*HSD17B12*)	0.08	−0.035	0.020	8.06 × 10^−2^	17 606	–	–
Discovery + replication (Stages 1 + 2)							
rs905938[C] at 1q22 (*DCST2*)	0.24	0.046	0.008	2.46 × 10^−8^	40 235	–	–
rs12545524[G] at 8q22.1 (near *GDF6*)	0.13	0.042	0.011	9.08 × 10^−5^	39 784	–	–
rs11037473[A] at 11p11.2 (nearest genes *TTC17*-*HSD17B12*)	0.07	−0.069	0.014	1.49 × 10^−6^	39 865	–	–

SNPs markers are identified according to their standard rs numbers (NCBI build 36). Independent novel SNPs with a strong suggestive effect in the discovery analysis on birth length are shown (*P* < 1 × 10^−6^). SNPs in loci that are known to be associated with adult height were excluded for replication efforts (adult height loci: *LCORL*, *PTCH1*, *GPR126* and *HMGA2*). MAF, minor allele frequency; SE, standard error. *β* reflects differences in standardized birth length per minor allele. *P* values are obtained from linear regression of each SNP against standardized birth length adjusted for sex and gestational age. We included both GWA and metabochip cohorts in our discovery analysis, rs905938 is on the metabochip, and rs12545524 and rs11037473 are not, this explains the differences in numbers (*n*). Derived inconsistency statistic *I*^2^ and *HetP* values reflect heterogeneity across discovery studies with the use of Cochran's *Q* tests.

### Functional analyses

We assessed common variants with deleterious functional implications in linkage disequilibrium (LD, *r*^2^ > 0.80) with rs905938 using HaploReg (8). There were no non-synonymous variants in LD with rs905938. We found three putative functional intronic variants in high LD with rs905938. Details are depicted in Supplementary Material, Table S4. Subsequently, we assessed whether variants in the identified locus were involved in the regulation of messenger RNA expression (eQTLs) in genome-wide expression datasets of lymphoblastoid cell lines (LCLs, *N* = 1830) (9,10). We found *cis* eQTLs [false discovery rate (FDR) < 1% account for all SNP-probe pairs that were within 1 Mb of each other) for transcripts of *PBXIP1*, *GBA* and *ADAM15*. Yet, rs905938 and the *cis* eQTL SNPs were not in perfect LD (*r*^2^ < 0.80, Supplementary Material, Table S5). Therefore, we cannot exclude that multiple independent effects arise from the same region of association.

### *DCST2* and growth phenotypes

We tested the associations of rs905938[C] with ‘fetal growth’ measures in the 1st, 2nd and 3rd trimester of pregnancy in the Generation R Study (*N* = 5756) (11), infant length at 1 year of age (range 6–18 months; *N* = 28 228) in the Early Growth Genetics (EGG) consortium (12), and adult height in the Genetic Investigation of Anthropometric Traits (GIANT) consortium (*N* = 127 513) (5). Rs905938[C] was not associated with ‘fetal growth’ measures, but was associated with infant length and adult height (*P* < 0.05; Table 2).

**Table 2:** Associations of rs905938[C] in *DCST*2 related to birth length with ‘fetal growth’ measures, infant length and adult height

	*β*	SE	*P*
Generation R: fetal growth (*N* = 5756)			
First trimester			
Crown-rump length (*n* = 1126)	0.003	0.045	0.952
Second trimester			
Femur length (*n* = 5361)	−0.035	0.023	0.129
Third trimester			
Femur length (*n* = 5532)	−0.015	0.022	0.490
EGG: infant length			
Infant length at 1 year of age (*N* = 28 228)	0.035	0.010	5.54 × 10^−4^
GIANT: adult height			
Adult height (*N* = 127 513)	0.024	0.006	1.45 × 10^−5^

rs905938 C-allele with a genome-wide significant effect on birth length is shown (*P* < 5 × 10^−8^) in relation to ‘fetal growth’ measures, infant length and adult height. SE, standard error. *β* reflects difference in standard deviation scores per minor allele.

### Known adult height loci in relation to birth and infant length

**Figure 4:**
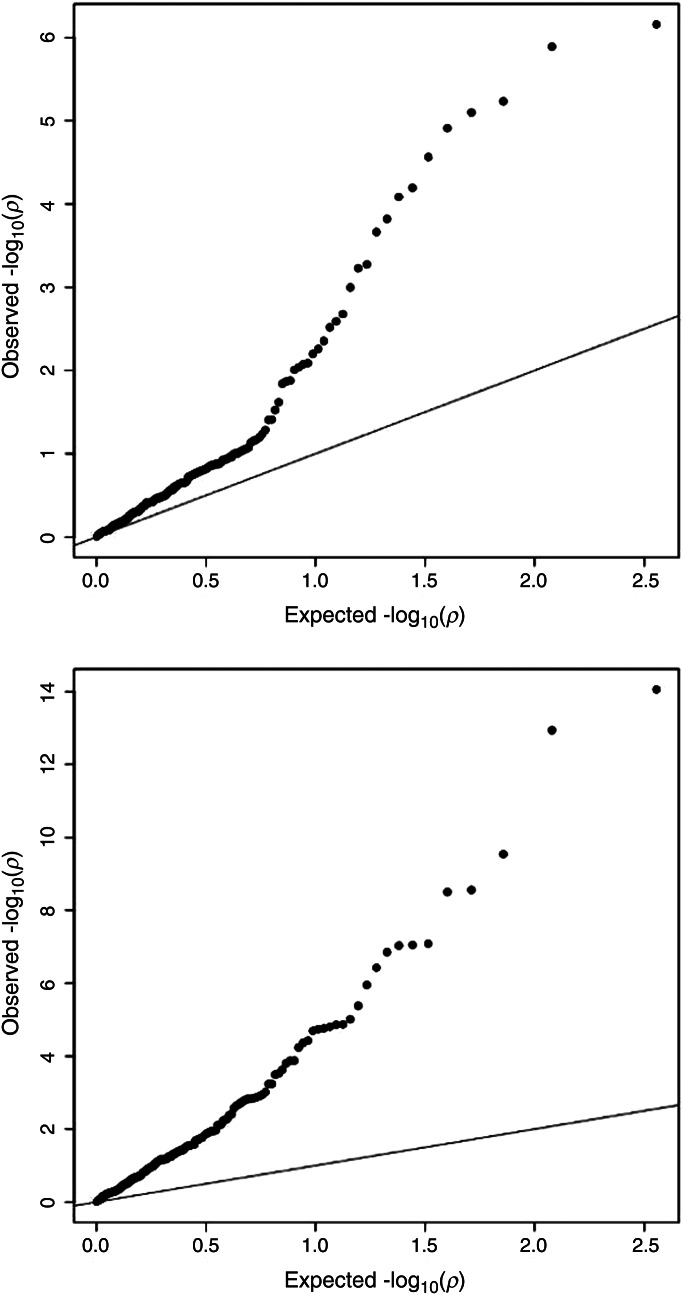
*QQ*-plots of the 180 known adult height SNPs with birth and infant length. *QQ*-plot of the 180 known adult height SNPs in association with birth length (upper panel) in 22 studies (*N* = 28 459) and with infant length (lower panel) in 19 studies (*N* = 28 238). The black dots represent observed *P* values and the diagonal lines represent the expected *P* values under the null distribution.

**Figure 5:**
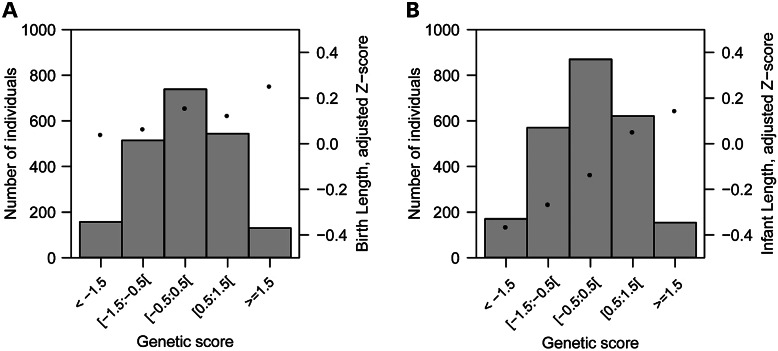
Height-increasing-alleles score of known adult height SNPs predicting birth and infant length. Genetic risk-allele scores (sum of height-increasing alleles weighted by known effect on adult height (5) transformed to standard deviation *Z*-scores) in the Generation R study plotted against length adjusted for sex and age. The distribution of the genetic risk-allele score is depicted as bars. (**A**) Mean birth length plotted against the genetic score (*N* = 2085). (**B**) Mean infant length plotted against the genetic score (*N* = 2385).

We also explored whether common genetic variants known to be associated with adult height (5) influenced birth length variation. We found that 17 out of 180 known adult height loci were associated with birth length (FDR < 5%, Supplementary Material, Table S6; Fig. 4, *QQ*-plot of 180 SNPs and birth length). We then calculated a height-increasing-alleles score of the 180 known height loci (5) to predict birth length in the Generation R Study (*N* = 2085; Fig. 5). The score composed of variants associated with adult height explained 0.13% of the variance in birth length (*P* = 0.1), in contrast to the ∼10% of the phenotypic variation in adult height reported in the original manuscript (5).

To evaluate whether different common genetic variants influenced both birth and infant length, we tested 2 193 675 million SNPs for association with infant length in almost the same set of samples used for the analysis of birth length (19 studies, *N* = 28 238; Supplementary Material, Table S7). We identified genome-wide significant associations at 11 genetic loci (Supplementary Material, Figs S3 and S4, *QQ*- and Manhattan plot), which all are known to be associated with adult height (Table 3, SNPs in or near *SF3B4*, *LCORL*, *SPAG17*, *C6orf173*, *PTCH1*, *GDF5*, *ZNFX1*, *HHIP*, *ACAN*, *HLA* locus and *HMGA2*) (5,13). In addition, we found that variants in 58 of the adult height loci were associated with infant length at an FDR of 5% (Supplementary Material, Table S8; Fig. 4, *QQ*-plot of 180 SNPs and infant length). Next, we tested in the Generation R Study (*N* = 2385) how much of the phenotypic variance in infant length was explained by the score composed of height-increasing-alleles. Variants from the 180 known adult height loci together explained 2.95% of the variance in infant length (*P* = 3.10 × 10^−17^, Fig. 5).

**Table 3:** Summary statistics of the eleven known adult height SNPs in association with infant length at *P* < 5 × 10^−8^

Marker	MAF	*β*	SE	*P*	*n*	*I*^2^	*HetP*
rs7536458[G] at 1p12 (*SPAG17*)	0.25	−0.064	0.010	9.61 × 10^−11^	28234	0.0	0.403
rs11205303[C] at 1q21.2 (*SF3B4*)	0.34	0.087	0.011	1.79 × 10^−16^	26559	0.0	0.864
rs1380294[T] at 4p15.31 (*LCORL*)	0.15	−0.108	0.014	2.54 × 10^−14^	23079	13.7	0.184
rs1812175[A] at 4q28-q32(*HHIP*)	0.18	−0.068	0.011	2.33 × 10^−9^	28227	0.0	0.398
rs592229[G] at (*HLA* locus)	0.43	0.048	0.009	2.22 × 10^−8^	28223	0.6	0.326
rs9385399[T] at 6q22.32 (*C6orf173*)	0.46	0.055	0.009	1.68 × 10^−10^	28224	0.0	0.943
rs1984119[C] at 9q22.3 (*PTCH1*)	0.26	−0.063	0.010	1.77 × 10^−10^	28197	0.0	0.490
rs7970350[T] at 12q15 (*HMGA2*)	0.49	−0.047	0.009	2.90 × 10^−8^	28226	0.0	0.426
rs2280470[A] at 15q26.1 (*ACAN*)	0.36	0.053	0.009	6.43 × 10^−9^	27443	0.0	0.436
rs143384[G] at 20q11.2 (*GDF5*)	0.44	0.058	0.009	2.87 × 10^−10^	28232	0.0	0.996
rs1567865[T] at 20q13.13 (*ZNFX1*)	0.21	0.063	0.010	1.10 × 10^−9^	28229	22.5	0.104

SNPs markers are identified according to their standard rs numbers (NCBI build 36). The total sample includes data of 19 independent datasets (*N* = 28 238). MAF, minor allele frequency; SE, standard error. *β* reflects differences in standardized infant length per minor allele. *P* values are obtained from linear regression of each SNP against standardized infant length adjusted for sex and age. We included both GWA and metabochip cohorts in our discovery analysis, this explains the differences in numbers (*n*). Derived inconsistency statistic *I*^2^ and *HetP* values reflect heterogeneity across discovery studies with the use of Cochran's *Q* tests.

### DEPICT analysis of birth and infant length

Finally, we used a pathway analysis tool called DEPICT (Pers *et al*., unpublished data) to prioritize genes at associated regions, search for reconstituted gene sets that were enriched in genes near associated variants, and identify tissue and cell types in which genes from loci associated with birth and infant length were highly expressed (full details in Materials and Methods). For both traits, we used independent SNPs (*r*^2^ < 0.05) associated at *P* < 1 × 10^−5^, from 21 birth length and 44 infant length loci. There were no pathways significantly overrepresented in the birth length results. In contrast, for infant length DEPICT significantly prioritized nine genes which were overrepresented (FDR < 5%, Supplementary Material, Table S9), including three known Mendelian human stature genes (*ACAN*, *GDF5* and *PTCH1*) as well as several relevant reconstituted gene sets (e.g. abnormal sternum ossification, regulation of osteoblast proliferation and WNT signaling, Supplementary Material, Table S10). There was no significant enrichment for particular tissue or cell types for any of the two traits.

## DISCUSSION

In the present study we identified one previously unknown locus (rs905938 in *DCST2* at 1q22) to be associated with birth length at a genome-wide significant level. This common genetic variant was also associated with infant length and adult height.

It was not possible to identify eQTLs for transcripts of *DCST2* in the MRCA and MRCE databases, as there were no probes available (9). Also, there was no significant eQTL of *DCST2* in immortalized LCLs (10). However, DCST2 is a DC-STAMP-like protein family member and DC-STAMP is an important regulator of osteoclast cell-fusion in bone homeostasis (14–16). The transcripts of *PBXIP1*, *GBA* and *ADAM15* were in weak LD with our lead SNP rs905938. The *PBXIP1* protein is known to regulate estrogen receptor functions (17). Mutations in the *GBA* gene cause Gaucher disease, and strong associations with Parkinson's disease and dementia with Lewy bodies have been described (18–21). *ADAM15* is prominently expressed in osteoblasts and to a lesser extent in osteoclasts (22). A study in mice showed that ADAM15 is required for normal skeletal homeostasis and that its absence causes increased nuclear translocation of β-catenin in osteoblasts leading to increased osteoblast proliferation and function, which results in higher trabecular and cortical bone mass (23). The 1q22 locus is a complex region harboring multiple interesting genes that could affect birth length. We emphasize that we could not specifically pinpoint the causal gene(s) as our lead SNP (rs905938) was not in perfect LD with our *cis* eQTL SNPs.

Although, there is some overlap between adult height loci and birth length, which is illustrated by 17 shared loci, the genetic architecture of adult height seems more similar to the genetic architecture of infant length than birth length [58 shared loci for infant length, based on conservative statistical method (FDR)]. One point of consideration for the interpretation of our findings is the potential of measurement error for birth length (24). This may lead to less power to detect novel genetic variants as standard errors of SNPs could be increased. The estimate of the risk-allele score slope of Figure 5 is not influenced by measurement error and the differences in the slopes suggest that birth and infant length are influenced by distinct genetic variants. We found that the SNP effects for birth length of 137 of the 180 established height loci were in the same direction as reported in the GIANT paper (5) (Supplementary Material, Table S6; probability of success = 0.761, *P* = 6.25 × 10^−13^). One hundred sixty-two of the 180 loci were in the same direction for infant length (Supplementary Material, Table S8; probability of success = 0.900, *P* = 2.20 × 10^−16^).

Four SNPs associated with birth length (*P* < 1 × 10^−5^) are in or near loci known to be associated with birth weight (*LCORL*, *HMGA2, ADCY5* and *ADRB1*). *LCORL* is associated with birth weight, birth length, infant length and adult height, but we could not find an obvious link between the gene and adult-onset diseases. *HMGA2* is associated with aortic root size (25), type 2 diabetes (26), and many other traits like tooth development, head circumference and brain structure (12,27). *ADCY5* is also associated with type 2 diabetes and *ADRB1* with adult blood pressure (2,3). These findings highlight genetic links between fetal growth and metabolism (2,3,26). As we found overlap between genetic variants of birth weight and birth length, we looked-up the effect of rs905938 in *DCST2* on birth weight in a previous EGG study (3)*.* Rs905938 was associated with birth weight, but weaker as compared with birth length (*β* = 0.035 SDS, SE = 0.010, *P* = 2.35 × 10^−4^, *N* = 26 558).

In conclusion, in the present study we identified one novel locus (rs905938 in *DCST2* at 1q22) associated with birth length at a genome-wide significant level. This common genetic variant was also associated with infant length and adult height, with decreasing magnitude of the associations in later life (0.046 SDS for birth length, 0.035 SDS for infant length and 0.024 SDS for adult height). To our knowledge, no phenotype has been previously associated with the *DCST2* gene and while the gene is expressed in osteoclasts, its function should be further studied.

## MATERIALS AND METHODS

### Stage 1: discovery genome-wide association analyses of birth length

We combined 21 population-based studies with GWA or Metabochip data and birth length available (total *N* = 28 459 individuals). One of our discovery cohorts had two independent sub-samples within their study leading to a total of 22 independent GWA/Metabochip sub-samples for our analysis: one sub-sample from the Avon Longitudinal Study of Parents and Children (ALSPAC, GWA, *n* = 4816); Children, Allergy, Milieu, Stockholm, Epidemiology [Swedish] (BAMSE, GWA, *n* = 423); Children's Hospital Of Philadelphia (CHOP, GWA, *n* = 432); Copenhagen Study on Asthma in Childhood 2000 (COPSAC-2000, GWA, *n* = 348); Copenhagen Study on Asthma in Childhood Registry (COPSAC-Registry, GWA, *n* = 1111); Danish National Birth Cohort (DNBC, GWA, *n* = 932); Generation R Study (Generation R, GWA, *n* = 2085); Hyperglycemia and Adverse Pregnancy Outcomes study (HAPO, GWA, *n* = 1325); Helsinki Birth Cohort Study (HBCS, GWA, *n* = 1572); Infancia y Medio Ambiente (INMA, GWA, *n* = 848); Leipzig Childhood Obesity cohort (LEIPZIG, Metbochip, *n* = 607); Lifestyle Immune System Allergy study (LISA, GWA, *n* = 552); Manchester Asthma and Allergy Study (MAAS, GWA, *n* = 402); Norwegian Mother and Child Cohort study (MOBA, GWA, *n* = 832); Northern Finland Birth Cohorts 1966 (NFBC66, GWA, *n* = 4642); Northern Finland Birth Cohorts 1986 (NFBC86, Metabochip, *n* = 4652); Physical Activity and Nutrition in Children study (PANIC, Metabochip, *n* = 319); two sub-samples from the Prevention and Incidence of Asthma and Mite Allergy birth cohort study (PIAMA1, GWA, *n* = 283; PIAMA2, GWA, *n* = 195); The Western Australian Pregnancy Cohort Study (RAINE, GWA, *n* = 1272); Special Turku Coronary Risk Factor Intervention Project (STRIP, Metabochip, *n* = 614); and TEENs of Attica: Genes and Environment (TEENAGE, GWA, *n* = 197). While no systematic phenotypic differences were observed between the sub-samples of the PIAMA birth cohort study, they were analyzed separately due to genotyping on different platforms and at different time periods. Genotypes within each study were obtained using high-density SNP arrays and then imputed for ∼2.5 M HapMap SNPs (Phase II, release 22; http://hapmap.ncbi.nlm.nih.gov/). The basic characteristics, exclusions applied (for example, individuals of non-European ancestry, family related individuals), genotyping, quality control and imputation methods for each discovery study are presented in Supplementary Material, Table S1.

#### Statistical analysis within discovery studies

In all studies, birth length was measured using standardized procedures. Studies with self-reported measurements were excluded a priori. Birth length was standardized using growth analyzer (http://www.growthanalyser.org), transforming birth length into sex- and age-adjusted SDS. We used the North-European 1991 reference panel to compare results between studies. Multiple births and twins were excluded from all analyses. We applied linear regression between number of alleles or dosages obtained from imputations and standardized birth length. The GWA analysis per study was performed using MaCH2qtl (28), SNPTEST (29), PLINK (30) or PropABEL (31). The secured data exchange and storage were facilitated by the Erasmus Medical Center, Department of Internal Medicine (32).

#### Meta-analysis of discovery studies

Prior to meta-analysis, SNPs with a MAF <0.01 and poorly imputed SNPs [r2hat <0.3 (MaCH); proper_info <0.4 (IMPUTE2); R2_BEALE <0.4 (BEAGLE)] were filtered. Genomic control (GC) (33) was applied to adjust the statistics generated within each cohort (see Supplementary Material, Table S1 for individual study *λ* values). Four out of the twenty-two sub-samples were genotyped on Metabochips. These SNP-arrays were enriched with ‘adult height SNPs'. Normal variation in early length growth seems to be associated with height in adulthood (1). Therefore, we assumed more true-positive hits in these studies and did not apply GC in these studies (GIANT *et al.*, unpublished data). Details of any additional corrections for study specific population structure are given in the Supplementary Material, Table S1. Inverse variance fixed-effects meta-analyses were analyzed using METAL (released 2010-08-01) (34) by two meta-analysts in parallel and blinded to obtain identical results. After the METAL meta-analysis, we filtered SNPs with a MAF <0.05 and SNPs that were not available in at least 12 sub-samples to avoid false-positive findings. We used Cochran's *Q* test and the derived inconsistency statistic *I*^2^ to assess evidence of between-study heterogeneity of the effect sizes. The meta-analysis results were obtained for a total of 2 201 971 SNPs. SNPs that crossed the threshold of *P ≤* 1 × 10^−6^ were considered to represent strong suggestive evidence of association with birth length. SNPs that were already known to be associated with adult height were excluded for the replication analysis (5). The explained variance of the top SNPs were calculated in one of the largest cohorts, the Generation R Study (*n* = 2085).

### Stage 2: replication analysis of top birth length SNPs

In the discovery phase, we found seven independent SNPs with strong suggestive evidence of association (*P* < 1 × 10^−6^) with birth length. Four SNPs were already known to be associated with adult height (5). These SNPs were excluded for follow-up analyses. The three remaining novel SNPs were followed-up in replication studies. We included both GWA and Metabochip studies in our discovery analysis. Rs905938 was on our Metabochips, and rs12545524 and rs11037473 were not. This results in differences in numbers for our top SNPs in the discovery and replication analyses. rs905938 was taken forward in 9 independent replication studies (*N* = 11 995), rs12545524 and rs11037473 in 13 independent replication studies including the four discovery Metabochip studies (*N* = 17 679). Details of the replication studies are presented in Supplementary Material, Table S2. Within the replication studies, we analyzed the association between number of alleles and standardized birth length. Combined effect estimates and heterogeneity between cohorts was calculated using fixed effects meta-analyses in R Version 2.8.1 (The R foundation for Statistical Computing, library rmeta). Top SNPs that crossed the significant threshold of *P*-replication ≤0.05 and the widely accepted genome-wide significance threshold of *P ≤* 5 × 10^−8^ for all studies combined were considered to represent robust evidence of association with birth length. The institutional review boards for human studies approved the protocols and written consent was obtained from the participating subjects or their caregivers if required by the institutional review board.

### DEPICT analysis

We used the novel Data-driven Expression-Prioritized Integration for Complex Traits (DEPICT) method (Pers *et al.,* unpublished data). DEPICT is designed to systematically identify the most likely causal gene at a given locus, gene sets that are enriched in genetic associations, and tissues and cell types in which genes from associated loci are highly expressed. First, DEPICT assigns genes to associated SNPs using LD *r*^2^ > 0.5 distance to define locus boundaries, merges overlapping loci and discards loci mapping within the extended major histocompatibility complex region (chromosome 6, base pairs 25 000–35 000). Next, the DEPICT method prioritizes genes within a given associated locus based on the genes' functional similarity to genes from other associated loci. Genes that are highly similar to genes from other loci obtain low prioritization *P* values, and simulated GWAS results are used to adjust for gene length bias as well as other potential confounders. There can be several prioritized genes in a given locus. Next, DEPICT conducts gene set enrichment analysis by testing whether genes in associated loci enrich for reconstituted versions of known pathways, gene sets as well as protein complexes. Leveraging the guilt by association hypothesis that genes co-expressing with genes from a given gene set are likely to be part of that gene set (see Cvejic *et al.* (35), for details), the gene set reconstitution is accomplished by identifying genes that were co-expressed with genes in a given gene set based on a panel of 77 840 gene expression microarrays. Gene sets from the following repositories were reconstituted: 5984 protein complexes that were derived from 169 810 high-confidence experimentally derived protein–protein interactions (36); 2473 phenotypic gene sets derived from 211 882 gene–phenotype pairs from the Mouse Genetics Initiative (37); 737 Reactome database pathways (38); 184 KEGG database pathways (39); and 5083 Gene Ontology database terms (40). Finally, DEPICT conducts tissue and cell type enrichment analysis, by testing whether genes in associated loci are highly expressed in any of 209 Medical Subject Heading annotations of 37 427 microarrays from the Affymetrix U133 Plus 2.0 Array platform (see Wood *et al.* (41) and Geller *et al.* (42) for previous applications of DEPICT). In this work, 21 autosomal SNPs for birth length and 44 autosomal SNPs for infant length were used as input to DEPICT resulting in 21 and 41 non-overlapping loci, respectively, that covered a total of 34 genes and 83 genes, respectively. The gene prioritization, gene set enrichment and tissue/cell type enrichment analyses were run using the default settings in DEPICT.

## SUPPLEMENTARY MATERIAL

Supplementary Material is available at *HMG* online.

## FUNDING

R.M.F. is supported by a Sir Henry Wellcome Postdoctoral Fellowship (Wellcome Trust grant 085541/Z/08/Z). T.H.P. is supported by The Danish Council for Independent Research Medical Sciences (FSS) The Alfred Benzon Foundation. B.F. is supported by an Oak Foundation fellowship. M.M. is a Wellcome Trust Senior Investigator (Wellcome Trust grant 090532) and a NIHR Senior Investigator. T.M.F. is supported by the European Research Council grant: SZ-245 50371-GLUCOSEGENES-FP7-IDEAS-ERC. F.R. (VIDI 016.136.367) and V.W.V.J. (VIDI 016.136.361) received grants from the Netherlands Organization for Health Research and Development. The other authors did not receive funding for this manuscript.

## Supplementary Material

Supplementary Data

## References

[1] Paternoster L., Howe L.D., Tilling K., Weedon M.N., Freathy R.M., Frayling T.M., Kemp J.P., Smith G.D., Timpson N.J., Ring S.M. (2011). Adult height variants affect birth length and growth rate in children. Hum. Mol. Genet..

[2] Freathy R.M., Mook-Kanamori D.O., Sovio U., Prokopenko I., Timpson N.J., Berry D.J., Warrington N.M., Widen E., Hottenga J.J., Kaakinen M. (2010). Variants in ADCY5 and near CCNL1 are associated with fetal growth and birth weight. Nat. Genet..

[3] Horikoshi M., Yaghootkar H., Mook-Kanamori D.O., Sovio U., Taal H.R., Hennig B.J., Bradfield J.P., St Pourcain B., Evans D.M., Charoen P. (2013). New loci associated with birth weight identify genetic links between intrauterine growth and adult height and metabolism. Nat. Genet..

[4] Mook-Kanamori D.O., van Beijsterveldt C.E., Steegers E.A., Aulchenko Y.S., Raat H., Hofman A., Eilers P.H., Boomsma D.I., Jaddoe V.W. (2012). Heritability estimates of body size in fetal life and early childhood. PLoS One.

[5] Lango Allen H., Estrada K., Lettre G., Berndt S.I., Weedon M.N., Rivadeneira F., Willer C.J., Jackson A.U., Vedantam S., Raychaudhuri S. (2010). Hundreds of variants clustered in genomic loci and biological pathways affect human height. Nature.

[6] Woods K.A., Camacho-Hubner C., Savage M.O., Clark A.J. (1996). Intrauterine growth retardation and postnatal growth failure associated with deletion of the insulin-like growth factor I gene. N. Engl. J. Med..

[7] Abuzzahab M.J., Schneider A., Goddard A., Grigorescu F., Lautier C., Keller E., Kiess W., Klammt J., Kratzsch J., Osgood D. (2003). IGF-I receptor mutations resulting in intrauterine and postnatal growth retardation. N. Engl. J. Med..

[8] Ward L.D., Kellis M. (2012). HaploReg: a resource for exploring chromatin states, conservation, and regulatory motif alterations within sets of genetically linked variants. Nucleic Acids Res..

[9] Liang L., Morar N., Dixon A.L., Lathrop G.M., Abecasis G.R., Moffatt M.F., Cookson W.O. (2013). A cross-platform analysis of 14 177 expression quantitative trait loci derived from lymphoblastoid cell lines. Genome Res..

[10] Granell R., Henderson A.J., Timpson N., St Pourcain B., Kemp J.P., Ring S.M., Ho K., Montgomery S.B., Dermitzakis E.T., Evans D.M. (2013). Examination of the relationship between variation at 17q21 and childhood wheeze phenotypes. J. Allergy Clin. Immunol.

[11] Jaddoe V.W., van Duijn C.M., Franco O.H., van der Heijden A.J., van Iizendoorn M.H., de Jongste J.C., van der Lugt A., Mackenbach J.P., Moll H.A., Raat H. (2012). The Generation R Study: design and cohort update 2012. Eur. J. Epidemiol.

[12] Taal H.R., St Pourcain B., Thiering E., Das S., Mook-Kanamori D.O., Warrington N.M., Kaakinen M., Kreiner-Moller E., Bradfield J.P., Freathy R.M. (2012). Common variants at 12q15 and 12q24 are associated with infant head circumference. Nat. Genet..

[13] Weedon M.N., Lango H., Lindgren C.M., Wallace C., Evans D.M., Mangino M., Freathy R.M., Perry J.R., Stevens S., Hall A.S. (2008). Genome-wide association analysis identifies 20 loci that influence adult height. Nat. Genet..

[14] Kukita T., Wada N., Kukita A., Kakimoto T., Sandra F., Toh K., Nagata K., Iijima T., Horiuchi M., Matsusaki H. (2004). RANKL-induced DC-STAMP is essential for osteoclastogenesis. J. Exp. Med..

[15] Yagi M., Miyamoto T., Sawatani Y., Iwamoto K., Hosogane N., Fujita N., Morita K., Ninomiya K., Suzuki T., Miyamoto K. (2005). DC-STAMP is essential for cell–cell fusion in osteoclasts and foreign body giant cells. J Exp Med.

[16] Jansen B.J., Eleveld-Trancikova D., Sanecka A., van Hout-Kuijer M., Hendriks I.A., Looman M.G., Leusen J.H., Adema G.J. (2009). OS9 interacts with DC-STAMP and modulates its intracellular localization in response to TLR ligation. Mol. Immunol..

[17] Manavathi B., Lo D., Bugide S., Dey O., Imren S., Weiss M.J., Humphries R.K. (2012). Functional regulation of pre-B-cell leukemia homeobox interacting protein 1 (PBXIP1/HPIP) in erythroid differentiation. J. Biol. Chem..

[18] Stone D.L., Tayebi N., Orvisky E., Stubblefield B., Madike V., Sidransky E. (2000). Glucocerebrosidase gene mutations in patients with type 2 Gaucher disease. Hum. Mutat..

[19] Sidransky E., Nalls M.A., Aasly J.O., Aharon-Peretz J., Annesi G., Barbosa E.R., Bar-Shira A., Berg D., Bras J., Brice A. (2009). Multicenter analysis of glucocerebrosidase mutations in Parkinson’s disease. N. Engl. J. Med..

[20] Chahine L.M., Qiang J., Ashbridge E., Minger J., Yearout D., Horn S., Colcher A., Hurtig H.I., Lee V.M., Van Deerlin V.M. (2013). Clinical and biochemical differences in patients having Parkinson disease with vs without GBA mutations. JAMA Neurol..

[21] Nalls M.A., Duran R., Lopez G., Kurzawa-Akanbi M., McKeith I.G., Chinnery P.F., Morris C.M., Theuns J., Crosiers D., Cras P. (2013). A multicenter study of glucocerebrosidase mutations in dementia with Lewy bodies. JAMA Neurol..

[22] Inoue D., Reid M., Lum L., Kratzschmar J., Weskamp G., Myung Y.M., Baron R., Blobel C.P. (1998). Cloning and initial characterization of mouse meltrin beta and analysis of the expression of four metalloprotease-disintegrins in bone cells. J. Biol. Chem..

[23] Marzia M., Guaiquil V., Horne W.C., Blobel C.P., Baron R., Chiusaroli R. (2011). Lack of ADAM15 in mice is associated with increased osteoblast function and bone mass. Biol. Chem..

[24] Johnson T.S., Engstrom J.L., Warda J.A., Kabat M., Peters B. (1998). Reliability of length measurements in full-term neonates. J. Obstet. Gynecol. Neonatal. Nurs..

[25] Vasan R.S., Glazer N.L., Felix J.F., Lieb W., Wild P.S., Felix S.B., Watzinger N., Larson M.G., Smith N.L., Dehghan A. (2009). Genetic variants associated with cardiac structure and function: a meta-analysis and replication of genome-wide association data. JAMA.

[26] Voight B.F., Scott L.J., Steinthorsdottir V., Morris A.P., Dina C., Welch R.P., Zeggini E., Huth C., Aulchenko Y.S., Thorleifsson G. (2010). Twelve type 2 diabetes susceptibility loci identified through large-scale association analysis. Nat. Genet..

[27] Ikram M.A., Fornage M., Smith A.V., Seshadri S., Schmidt R., Debette S., Vrooman H.A., Sigurdsson S., Ropele S., Taal H.R. (2012). Common variants at 6q22 and 17q21 are associated with intracranial volume. Nat. Genet..

[28] Li Y., Willer C.J., Ding J., Scheet P., Abecasis G.R. (2010). MaCH: using sequence and genotype data to estimate haplotypes and unobserved genotypes. Genet. Epidemiol..

[29] Marchini J., Howie B., Myers S., McVean G., Donnelly P. (2007). A new multipoint method for genome-wide association studies by imputation of genotypes. Nat. Genet..

[30] Purcell S., Neale B., Todd-Brown K., Thomas L., Ferreira M.A., Bender D., Maller J., Sklar P., de Bakker P.I., Daly M.J. (2007). PLINK: a tool set for whole-genome association and population-based linkage analyses. Am. J. Hum. Genet..

[31] Aulchenko Y.S., Struchalin M.V., van Duijn C.M. (2010). ProbABEL package for genome-wide association analysis of imputed data. BMC Bioinformatics.

[32] Estrada K., Abuseiris A., Grosveld F.G., Uitterlinden A.G., Knoch T.A., Rivadeneira F. (2009). GRIMP: a web- and grid-based tool for high-speed analysis of large-scale genome-wide association using imputed data. Bioinformatics.

[33] Devlin B., Roeder K. (1999). Genomic control for association studies. Biometrics.

[34] Willer C.J., Li Y., Abecasis G.R. (2010). METAL: fast and efficient meta-analysis of genomewide association scans. Bioinformatics.

[35] Cvejic A., Haer-Wigman L., Stephens J.C., Kostadima M., Smethurst P.A., Frontini M., van den Akker E., Bertone P., Bielczyk-Maczynska E., Farrow S. (2013). SMIM1 underlies the Vel blood group and influences red blood cell traits. Nat. Genet..

[36] Lage K., Karlberg E.O., Storling Z.M., Olason P.I., Pedersen A.G., Rigina O., Hinsby A.M., Tumer Z., Pociot F., Tommerup N. (2007). A human phenome–interactome network of protein complexes implicated in genetic disorders. Nat. Biotechnol..

[37] Bult C.J., Richardson R.J., Blake J.A., Kadin J.A., Ringwald M., Eppig J.T. (2000). Mouse genome informatics in a new age of biological inquiry.

[38] Croft D., O'Kelly G., Wu G., Haw R., Gillespie M., Matthews L., Caudy M., Garapati P., Gopinath G., Jassal B. (2011). Reactome: a database of reactions, pathways and biological processes. Nucleic Acids Res..

[39] Kanehisa M., Goto S., Sato Y., Furumichi M., Tanabe M. (2012). KEGG for integration and interpretation of large-scale molecular data sets. Nucleic Acids Res..

[40] Ashburner M., Ball C.A., Blake J.A., Botstein D., Butler H., Cherry J.M., Davis A.P., Dolinski K., Dwight S.S., Eppig J.T. (2000). Gene ontology: tool for the unification of biology. The Gene Ontology Consortium. Nat. Genet..

[41] Wood A.R., Esko T., Yang J., Vedantam S., Pers T.H., Gustafsson S., Chu A.Y., Estrada K., Luan J., Kutalik Z. (2014). Defining the role of common variation in the genomic and biological architecture of adult human height. Nat. Genet..

[42] Geller F., Feenstra B., Carstensen L., Pers T.H., van Rooij I.A., Korberg I.B., Choudhry S., Karjalainen J.M., Schnack T.H., Hollegaard M.V. (2014). Genome-wide association analyses identify variants in developmental genes associated with hypospadias. Nat. Genet.

[43] Hocher B., Chen Y.P., Schlemm L., Burdack A., Li J., Halle H., Pfab T., Kalk P., Lang F., Godes M. (2009). Fetal sex determines the impact of maternal PROGINS progesterone receptor polymorphism on maternal physiology during pregnancy. Pharmacogenet Genomics.

[44] Pfab T., Slowinski T., Godes M., Halle H., Priem F., Hocher B (2006). Low birth weight, a risk factor for cardiovascular diseases in later life, is already associated with elevated fetal glycosylated hemoglobin at birth. Circulation.

[45] Hocher B., Slowinski T., Stolze T., Pleschka A., Neumayer HH., Halle H. (2000). Association of maternal G protein beta3 subunit 825T allele with low birthweight. Lancet.

