# Supplementary material for: A novel common variant in DCST2 is associated with length in early life and height in adulthood
Source: Hum Mol Genet. 2014 Oct 3;24(4):1155–68. doi: 10.1093/hmg/ddu510 (PMC4447786; doi:10.1093/hmg/ddu510)
Supplement: Supplementary Data [file supp_24_4_1155__index.html]

A novel common variant in DCST2 is associated with length in early life and height in adulthood — A novel common variant in DCST2 is associated with length in early life and height in adulthood — Supplementary Data 

# A novel common variant in *DCST2* is associated with length in early life and height in adulthood

## Supplementary Data

Supplementary Data

**Files in this Data Supplement:**

- Supplementary Data - Pdf file
- Supplementary Tables - xls file
