# Supplementary material for: A novel common variant in DCST2 is associated with length in early life and height in adulthood
Source: Hum Mol Genet. 2014 Oct 3;24(4):1155–68. doi: 10.1093/hmg/ddu510 (PMC4447786; doi:10.1093/hmg/ddu510)
Supplement: Supplementary Data [file supp_ddu510_ddu510supp.pdf]

A novel common variant in *DCST2* is associated with length in early life and height in adulthood

SUPPLEMENTAL DATA

SUPPLEMENTAL DATA FIGURE LEGENDS

Figure S1. QQ-plot of 2,201,971 SNPs from the 22 birth length discovery studies (N = 28,459).

The black dots represent observed *P* values and the red line represents the expected *P* values under the null distribution.

Figure S2. Manhattan plot of 2,201,971 SNPs from the 22 birth length discovery studies (N = 28,459).

The $-\log_{10}$ of association *P* values for each SNP (y-axis) is plotted against the genomic position (x-axis). The red line represents genome-wide significance level.

Figure S3. QQ-plot of 2,193,675 SNPs from the 19 infant length studies (N = 28,238).

The black dots represent observed *P* values and the red line represents the expected *P* values under the null distribution.

Figure S4. Manhattan plot of 2,193,675 SNPs from the 19 infant length studies (N = 28,238).

The $-\log_{10}$ of association *P* values for each SNP (y-axis) is plotted against the genomic position (x-axis). The red line represents genome-wide significance level.

Figure S1. QQ-plot of 2,201,971 SNPs from the 22 birth length discovery studies (N = 28,459).

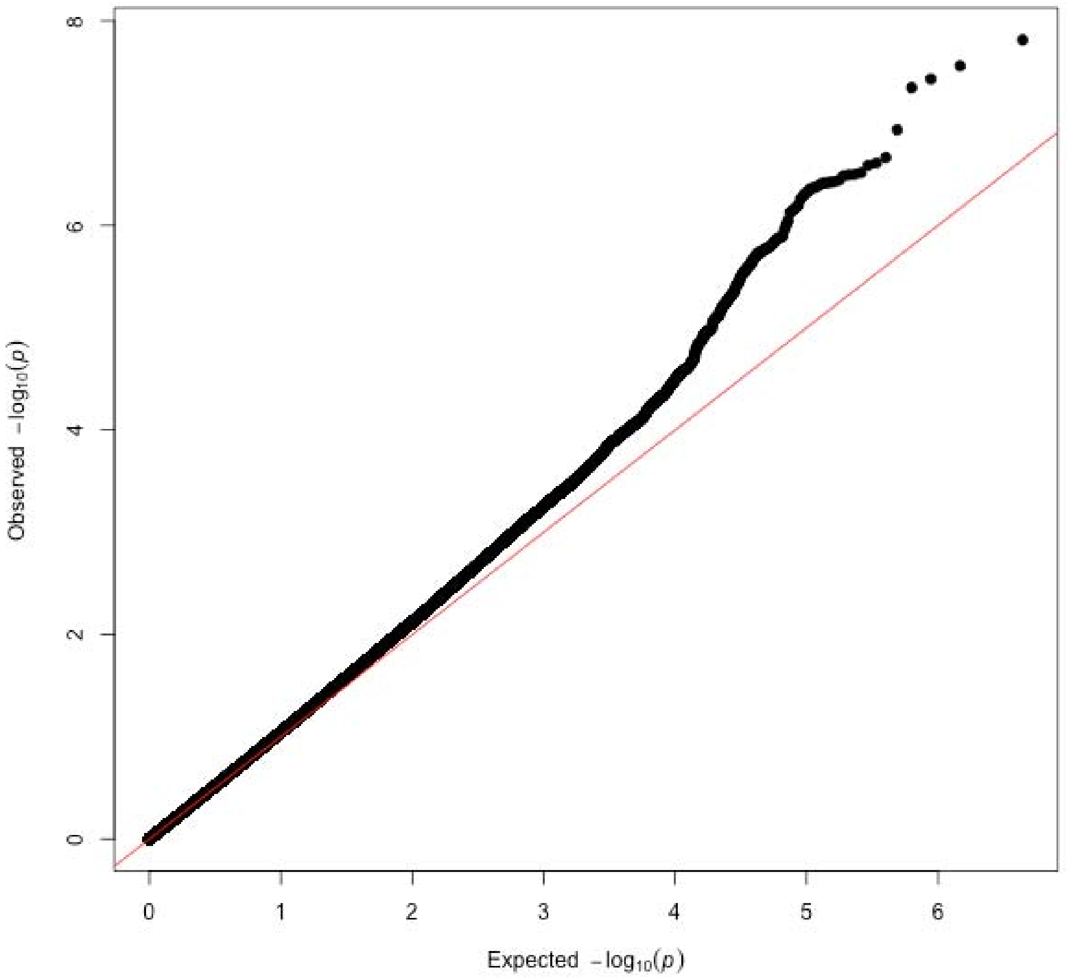

Figure S2. Manhattan plot of 2,201,971 SNPs from the 22 birth length discovery studies (N = 28,459).

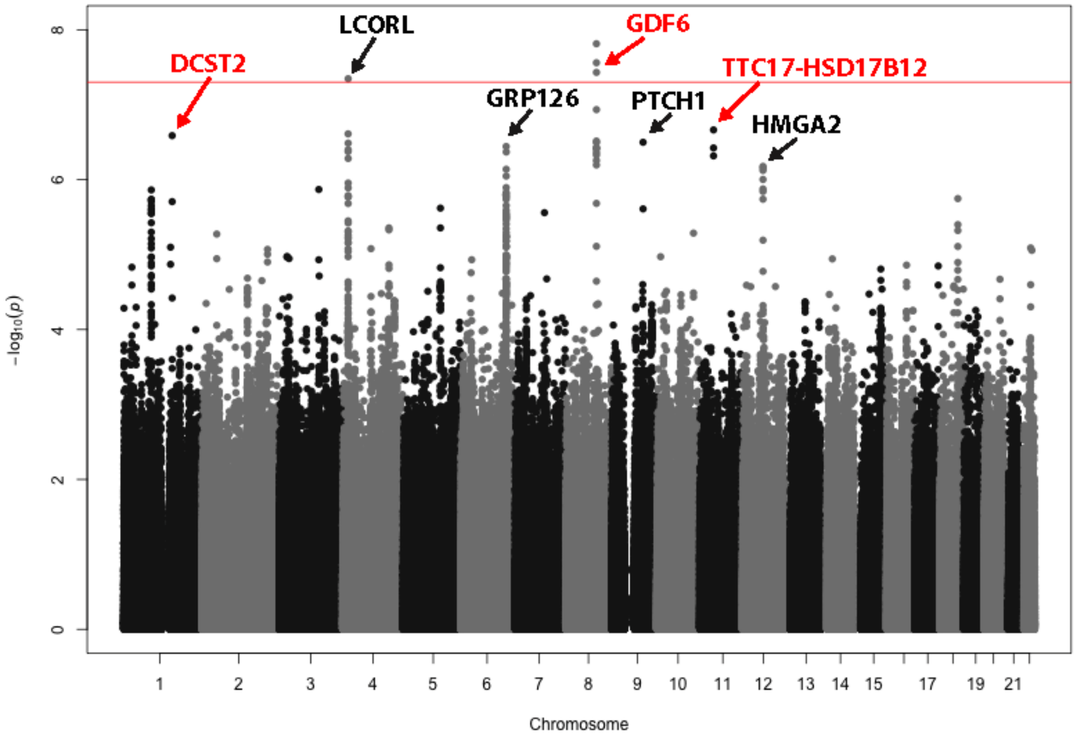

Figure S3. QQ-plot of 2,193,675 SNPs from the 19 infant length studies (N = 28,238).

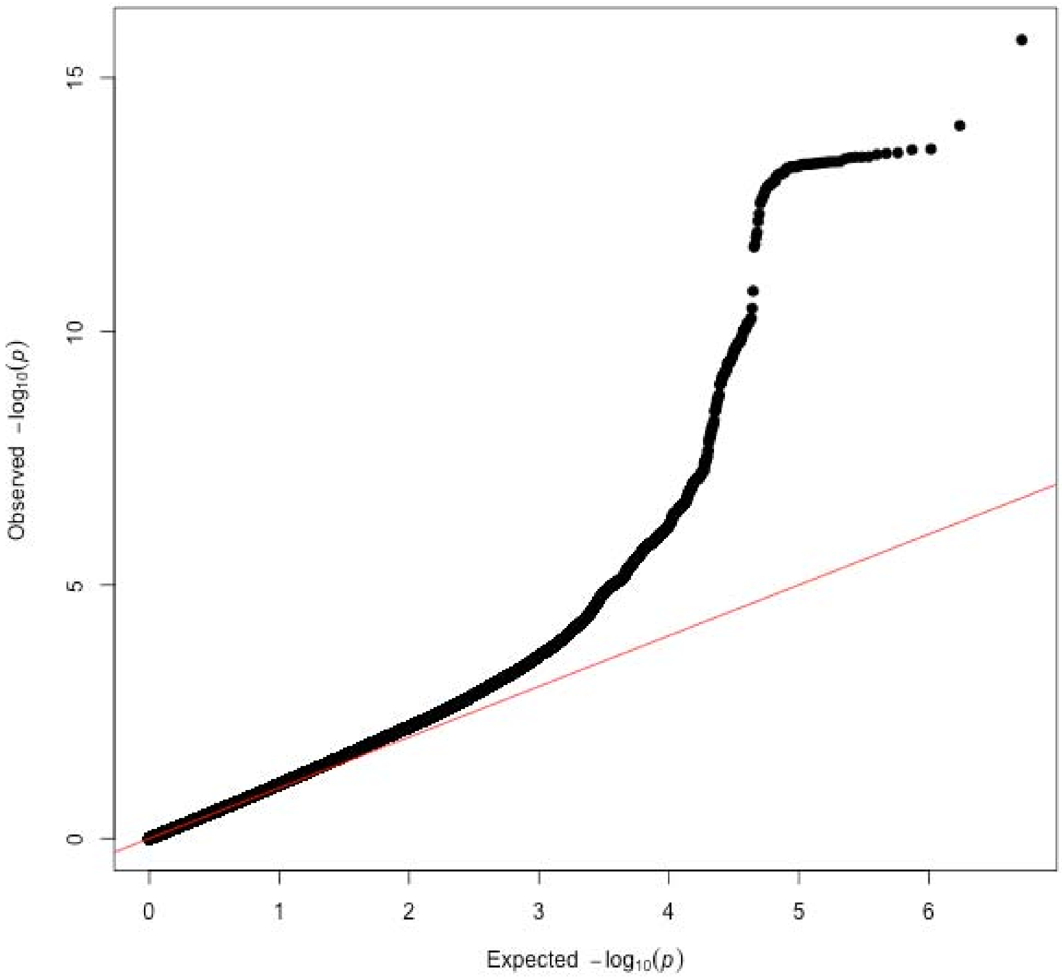

Figure S4. Manhattan plot of 2,193,675 SNPs from the 19 infant length studies (N = 28,238).

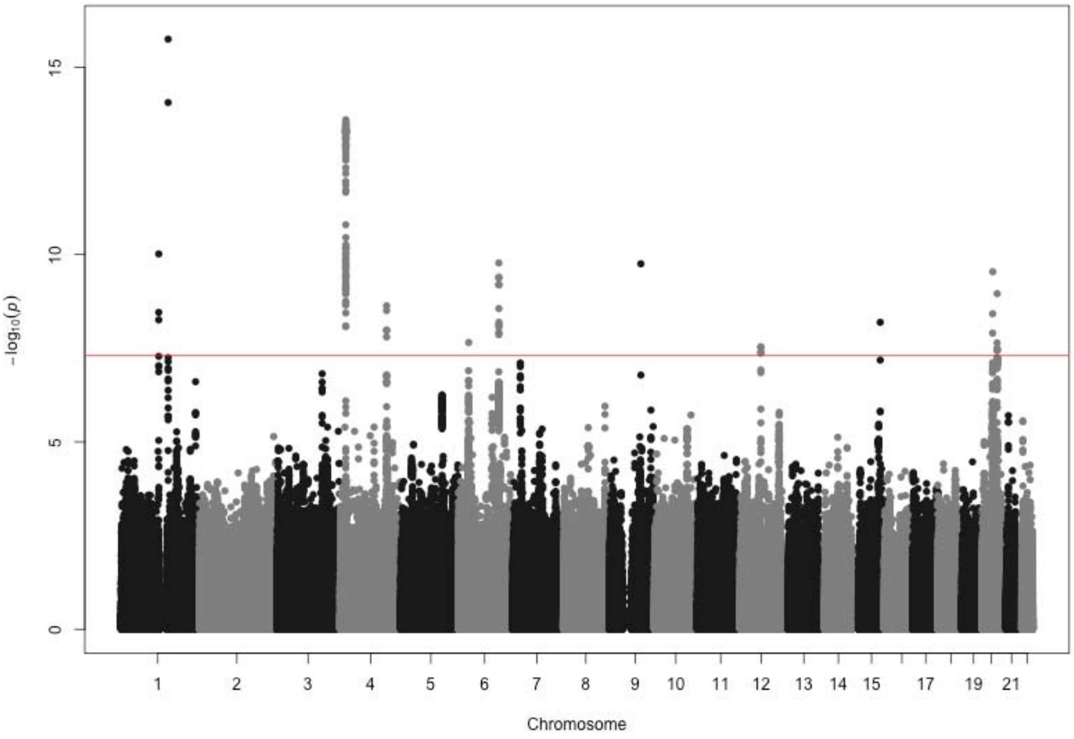

Table S3. Summary statistics of the 21 independently associated SNPs with birth length at $P < 1x10^{-5}$ (N = 28,459).

**Marker[effect allele]**	**MAF**	***ß***	**S.E.**	***P***	I^2	***HetP***	**Direction**	**n**
rs1576672[T] at 1:87695013 (*LMO4*)	0.47	-0.05	0.01	1.4E-06	0	9.1E-01	------??--?----??+--?+	18839
rs11205277[A] at 1:148159496 (*SF3B4*)	0.37	-0.04	0.01	8.0E-06	0	6.5E-01	-++---+---++----+----+	28325
rs905938[T] at 1:153258013 (*DCST2*)*	0.24	-0.05	0.01	2.6E-07	0	9.3E-01	-+-++------+------+--+	28327
rs17034876[T] at 2:46337814 (*EPAS1*)	0.30	0.05	0.01	5.3E-06	0	7.2E-01	+++-++++++?-+++??+-+?+	22140
rs10185378[T] at 2:206013401 (*PARD3B*)	0.06	0.09	0.02	8.5E-06	0	8.6E-01	+-++-+++-+?+-++??+++?+	22147
rs6798189[A] at 3:124578002 (*ADCY5*)	0.22	0.05	0.01	1.4E-06	0	7.1E-01	++++-++++++++++++++++-	28292
rs724577[A] at 4:17602508 (*LCORL*)	0.29	0.05	0.01	4.5E-08	7.2	3.6E-01	+++-+++++++-+-++-++-+-	28332
rs2904185[T] at 4:89358856 (*PPM1K*)	0.45	0.04	0.01	8.3E-06	0	6.6E-01	++-+++-+++?++++??+-+?+	22143
rs13146972[T] at 4:145789142 (*HHIP*)	0.45	0.04	0.01	4.4E-06	0	8.4E-01	+++++++++++--++++++++-	28447
rs9327035[T] at 5:116563098	0.40	0.05	0.01	2.4E-06	26.1	1.5E-01	+-+-++++++?-+++??+++?+	21758
rs3011890[T] at 6:141492122	0.27	-0.05	0.01	1.6E-06	16.6	2.4E-01	----+---------+--++--+	28300
rs6570507[A] at 6:142721265 (*GPR126*)	0.29	-0.05	0.01	3.6E-07	3.3	4.2E-01	---+--+---+-----++----	28339
rs13229771[T] at 7:92369727 (*CDK6*)	0.13	0.07	0.01	2.8E-06	42.4	3.0E-02	+---++++-+?-+++??-+-?-	22128
rs12545524[A] at 8:96974821 (*GDF6*)*	0.14	-0.08	0.01	1.5E-08	6.6	3.8E-01	---------+?----??---?-	22170
rs1984119[T] at 9:97408582 (*PTCH1*)	0.27	0.05	0.01	3.2E-07	0	5.4E-01	+--+++-+++++-+++++-++-	28329
rs740746[A] at 10:115782777 (*ADRB1*)	0.26	0.04	0.01	5.2E-06	0	8.6E-01	++++++-++-+++++++++-++	28332
rs11037473[A] at 11:43532643 (*TTC17-HSD17B12*)*	0.06	-0.11	0.02	2.2E-07	0	7.3E-01	--------++?----??-+-?+	22259

rs1042725[T] at 12:64644614 (*HMGA2*)	0.49	-0.04	0.01	6.7E-07	20.2	1.9E-01	-+---+--++-+-----+----	28442
rs12967139[A] at 18:57138211 (*CDH20*)	0.32	0.05	0.01	1.8E-06	42	3.2E-02	++-+++++++?++++??-++?+	22265
rs16985662[A] at 22:36956224 (*TMEM184B*)	0.18	-0.06	0.01	8.1E-06	0	5.1E-01	--+-+---+-?+---??---?+	22209
rs10483213[A] at 22:40669471 (*CENOM*)	0.10	-0.06	0.01	8.8E-06	21.9	1.7E-01	---++----+--+----++--+	28313

Single nucleotide polymorphisms (SNPs) markers are identified according to their standard rs numbers (NCBI build 36). Independent SNPs with a suggestive effect in the discovery analysis on birth length are shown ($P < 1x10^{-5}$). The total sample includes data of 22 independent datasets (N = 28,459). *: SNPs taken forward for replication. MAF, minor allele frequency; S.E., standard error. *ß* reflects differences in standardized birth length per effect allele. *P* values are obtained from linear regression of each SNP against standardized birth length adjusted for sex and gestational age. Derived inconsistency statistic I^2 and *HetP* values reflect heterogeneity across discovery studies with the use of Cochran's Q tests. Direction of effect per study (order of datasets: ALSPAC, BAMSE, CHOP, COPSAC-2000, COPSAC-REGISTRY, DNBC, GENR, HAPO, HBCS, INMA, LEIPZIG, LISA, MAAS, MOBA, NFBC66, NFBC86, PANIC, PIAMA1, PIAMA2, RAINE, STRIP and TEENAGE). We included both GWA and metabochip cohorts in our discovery analysis, this explains the differences in numbers (n).

Table S4. HaploReg search for functional variants in LD with rs905938.

Marker	Position	r^2	D’	Enhancer histone marks	Proteins bound	Motifs changed	GENCODE	Function
rs905938	154991389	NA	NA	4 cell types	-	5 altered motifs	*DCST2*	Intronic
rs1870940	154984363	0.89	0.95	4 cell types	EBF1	RXRA,TFIIA	*ZBTB7B*	Intronic
rs112439021	154993915	0.99	1.00	-	-	6 altered motifs	*DCST2*	Intronic
rs76798800	154994978	0.97	1.00	4 cell types	AP2ALPHA, AP2GAMMA	LRH1	*DCST2*	Intronic

The functional annotated SNPs in LD with rs905938 were not available in HapMap release 22 and therefore we could not show the association between the annotated makers and birth length. NA, not applicable.

Table S5. eQTLs in LCLs of SNPs in LD with rs905938 (N = 1,830).

Peak marker	Distance	D’	r^2	H^2	*P*	n	Transcript	Dataset
rs6674853	98636	0.565	0.083	20.7%	3.21×10^{-16}	405	*PBXIP1*	MRCA
rs12407919	207926	0.137	0.014	10.1%	1.28×10^{-11}	550	*GBA*	MRCE
rs6673081	1794	1.000	0.509	2.1%	1.86×10^{-5}	875	*ADAM15*	ALSPAC

eQTLs, expression quantitative trait loci; LCLs, lymphoblastoid cell lines; LD, linkage disequilibrium. Genes within 1Mb of rs905938. H^2 is the % variance in expression after adjusting for batch effects explained by SNP. *P* values are obtained from a score test taking into account the family relatedness of the sample. The *PBXIP1* protein is known to regulate estrogen receptor functions(1). Mutations in the *GBA* gene cause Gaucher disease, and strong associations with Parkinson’s disease and dementia with Lewy bodies have been described(2-5). *ADAM15* is prominently expressed in osteoblasts and to a lesser extent in osteoclasts(6). A study in mice showed that ADAM15 is required for normal skeletal homeostasis and that its absence causes increased nuclear translocation of β-catenin in osteoblasts leading to increased osteoblast proliferation and function, which results in higher trabecular and cortical bone mass(7).

Table S6. Summary statistics of the known adult height loci and their association with birth length (N = 28,459).

Marker[effect allele]	MAF	ß	S.E.	*P*	*P*_FDR	*HetP*	n	GIANT
rs1351394[T] at 12:64638093 (HMGA2)	0.498	0.044	0.009	7.0E-07	**1.2E-04**	4.5E-01	27574	Same
rs6449353[T] at 4:17642586 (LCORL)*	0.141	0.065	0.013	1.3E-06	**1.2E-04**	5.8E-01	28442	Same
rs7763064[A] at 6:142838982 (GPR126)	0.288	-0.044	0.010	5.9E-06	**3.5E-04**	2.8E-01	28401	Same
rs11205277[A] at 1:148159496 (SF3B4)	0.373	-0.041	0.009	8.0E-06	**3.6E-04**	6.5E-01	28325	Same
rs1257763[A] at 9:95933766 (PTPDC1)	0.042	0.114	0.026	1.2E-05	**4.4E-04**	9.5E-01	26301	Same
rs7689420[T] at 4:145787802 (HHIP)	0.173	-0.048	0.012	2.7E-05	**8.2E-04**	7.3E-01	28451	Same
rs11259936[A] at 15:82371586 (ADAMTSL3)	0.481	-0.035	0.009	6.4E-05	**1.6E-03**	9.5E-01	28446	Same
rs724016[A] at 3:142588260 (ZBTB38)	0.453	-0.034	0.009	8.2E-05	**1.9E-03**	5.1E-01	28419	Same
rs2780226[T] at 6:34307070 (HMGA1)	0.076	-0.069	0.018	1.5E-04	**3.0E-03**	2.8E-01	25045	Same
rs2871865[C] at 15:97012419 (IGF1R)	0.111	0.065	0.018	2.2E-04	**3.9E-03**	5.7E-01	21959	Same
rs473902[T] at 9:97296056 (PTCH1)	0.084	0.064	0.018	5.3E-04	**8.7E-03**	2.6E-01	27878	Same
rs143384[A] at 20:33489170 (GDF5)	0.434	-0.032	0.009	5.9E-04	**8.9E-03**	7.7E-01	27807	Same
rs10863936[A] at 1:210304421 (DTL)	0.475	-0.028	0.009	1.0E-03	**1.4E-02**	4.4E-01	28308	Same
rs1708299[A] at 7:28156471 (JAZF1)	0.302	0.033	0.011	2.1E-03	**2.7E-02**	1.2E-01	22941	Same
rs4986172[T] at 17:40571807 (ACBD4)	0.364	-0.027	0.009	2.6E-03	**3.1E-02**	9.9E-01	27209	Same
rs3110496[A] at 17:24941897 (ANKRD13B)	0.361	-0.026	0.009	3.0E-03	**3.4E-02**	3.8E-01	28324	Same
rs5742915[T] at 15:72123686 (PML)	0.435	-0.025	0.009	4.4E-03	**4.7E-02**	2.2E-01	28324	Same

rs806794[A] at 6:26308656 (Histone cluster)	0.346	0.026	0.009	5.5E-03	5.5E-02	4.3E-01	27603	Same
rs2145272[A] at 20:6574218 (BMP2)	0.327	-0.026	0.009	6.3E-03	6.0E-02	3.9E-01	27622	Same
rs12534093[A] at 7:23469499 (IGF2BP3)	0.224	-0.033	0.012	8.2E-03	7.2E-02	4.2E-01	22132	Same
rs2247341[A] at 4:1671115 (SLBP)	0.385	0.024	0.009	8.4E-03	7.2E-02	4.5E-01	28412	Same
rs9428104[A] at 1:118657110 (SPAG17)	0.242	-0.030	0.012	9.2E-03	7.5E-02	7.2E-01	22234	Same
rs1173727[T] at 5:32866278 (NPR3)	0.398	0.026	0.010	9.8E-03	7.7E-02	3.8E-01	22259	Same
rs494459[T] at 11:118079885 (TREH)	0.410	0.021	0.009	1.3E-02	9.8E-02	5.8E-01	28332	Same
rs6470764[T] at 8:130794847 (GSDMC)	0.197	-0.028	0.011	1.4E-02	9.8E-02	2.6E-01	28448	Same
rs1043515[A] at 17:34175722 (PIP4K2B)	0.460	-0.022	0.009	1.4E-02	1.0E-01	4.6E-01	28319	Same
rs961764[C] at 6:117628849 (VGLL2)	0.419	-0.023	0.010	2.4E-02	1.6E-01	8.2E-01	22250	Same
rs6473015[A] at 8:78341040 (PEX2)	0.257	-0.021	0.010	3.0E-02	1.9E-01	8.5E-02	28332	Same
rs1582931[A] at 5:122685098 (CEP120)	0.498	-0.022	0.011	3.9E-02	2.4E-01	5.2E-02	22230	Same
rs6569648[T] at 6:130390812 (L3MBTL3)	0.225	-0.021	0.010	3.9E-02	2.4E-01	2.8E-01	28334	Same
rs2237886[T] at 11:2767307 (KCNQ1)	0.095	0.029	0.015	5.2E-02	3.0E-01	8.5E-01	27224	Same
rs16942341[T] at 15:87189909 (ACAN)	0.025	-0.060	0.032	5.8E-02	3.3E-01	5.3E-02	25508	Same
rs4821083[T] at 22:31386341 (SYN3)	0.153	0.023	0.012	6.4E-02	3.5E-01	1.8E-02	28440	Same
rs422421[T] at 5:176449932 (FGFR4/NSD1)	0.199	-0.021	0.011	6.8E-02	3.6E-01	2.4E-02	28310	Same
rs42235[T] at 7:92086012 (CDK6)	0.293	0.019	0.011	7.0E-02	3.6E-01	6.0E-01	23363	Same
rs1490384[T] at 6:126892853 (C6orf173)	0.496	0.016	0.009	7.3E-02	3.7E-01	8.9E-01	28119	Same
rs7178424[T] at 15:60167551 (C2CD4A)	0.459	-0.015	0.009	8.5E-02	4.1E-01	5.6E-01	28333	Same

rs7926971[A] at 11:12654616 (TEAD1)	0.456	-0.017	0.010	8.8E-02	4.2E-01	3.7E-01	22221	Same
rs7155279[T] at 14:91555634 (TRIP11)	0.364	-0.018	0.011	9.0E-02	4.2E-01	9.4E-02	22231	Same
rs862034[A] at 14:74060499 (LTBP2)	0.353	-0.018	0.011	9.4E-02	4.2E-01	4.0E-01	21375	Same
rs2336725[T] at 3:53093779 (RTF1)	0.430	-0.014	0.009	9.8E-02	4.2E-01	2.0E-01	28330	Same
rs1325598[A] at 1:175058872 (PAPPA2)	0.444	-0.017	0.010	9.9E-02	4.2E-01	7.5E-01	21417	Same
rs6879260[T] at 5:179663620 (GFPT2)	0.398	-0.015	0.009	1.0E-01	4.2E-01	7.7E-01	28422	Same
rs2284746[C] at 1:17179262 (MFAP2)	0.500	-0.017	0.010	1.1E-01	4.4E-01	2.5E-01	21266	Same
rs2597513[T] at 3:13530836 (HDAC11)	0.095	0.024	0.015	1.1E-01	4.4E-01	4.9E-02	26380	Opposite
rs798489[T] at 7:2768329 (GNA12)	0.307	-0.015	0.009	1.1E-01	4.4E-01	9.5E-01	28358	Same
rs7507204[C] at 19:3379834 (NFIC)	0.246	0.021	0.014	1.2E-01	4.4E-01	4.6E-01	17745	Same
rs2093210[T] at 14:60027032 (SIX6)	0.416	-0.017	0.011	1.2E-01	4.4E-01	8.8E-01	22250	Same
rs11867479[T] at 17:65601802 (KCNJ16)	0.325	0.014	0.009	1.3E-01	4.6E-01	4.2E-01	28331	Same
rs1659127[A] at 16:14295806 (MKL2)	0.332	-0.015	0.010	1.3E-01	4.6E-01	1.5E-01	28357	Opposite
rs9472414[A] at 6:45054484 (SUPT3H/RUNX2)	0.214	-0.018	0.012	1.3E-01	4.6E-01	5.3E-01	22259	Same
rs6457821[A] at 6:35510783 (PPARD/FANCE)	0.022	-0.062	0.041	1.3E-01	4.6E-01	9.4E-01	16598	Same
rs7466269[A] at 9:132453905 (FUBP3)	0.392	0.013	0.009	1.4E-01	4.6E-01	9.9E-01	28315	Same
rs2154319[T] at 1:41518357 (SCMH1)	0.239	-0.019	0.013	1.4E-01	4.6E-01	1.9E-01	21349	Same
rs1950500[T] at 14:23900690 (NFATC4)	0.286	0.014	0.009	1.4E-01	4.6E-01	2.3E-01	28333	Same
rs7849585[T] at 9:138251691 (QSOX2)	0.342	0.016	0.011	1.5E-01	4.7E-01	1.8E-01	21100	Same
rs1013209[T] at 8:24172249 (ADAM28)	0.260	-0.017	0.012	1.5E-01	4.8E-01	3.7E-01	22253	Same

rs891088[A] at 19:7135762 (INSR)	0.277	-0.014	0.010	1.5E-01	4.8E-01	2.4E-01	28326	Same
rs11830103[A] at 12:122389499 (SBNO1)	0.211	-0.019	0.014	1.6E-01	4.8E-01	1.8E-01	18023	Same
rs4072910[C] at 19:8550031 (ADAMTS10)	0.440	-0.016	0.012	1.6E-01	4.8E-01	4.0E-01	21131	Same
rs1046943[A] at 6:109890634 (ZBTB24)	0.437	0.014	0.010	1.6E-01	4.8E-01	8.7E-01	22248	Same
rs6684205[A] at 1:216676325 (TGFB2)	0.294	0.014	0.010	1.6E-01	4.8E-01	2.1E-01	23686	Opposite
rs10748128[T] at 12:68113925 (FRS2)	0.353	0.013	0.010	1.7E-01	4.8E-01	8.3E-01	24918	Same
rs7460090[T] at 8:57356717 (SDR16C5)	0.128	0.021	0.015	1.7E-01	4.8E-01	1.7E-01	22208	Same
rs3791675[T] at 2:55964813 (EFEMP1)	0.237	-0.014	0.010	1.8E-01	4.9E-01	3.6E-01	28433	Same
rs1814175[T] at 11:49515748 (FOLH1)	0.379	0.013	0.009	1.8E-01	4.9E-01	5.8E-01	26614	Same
rs2629046[T] at 2:224755988 (SERPINE2)	0.449	0.013	0.010	1.8E-01	4.9E-01	5.6E-01	22237	Same
rs10010325[A] at 4:106325802 (TET2)	0.463	0.011	0.008	1.9E-01	4.9E-01	2.6E-01	28316	Same
rs237743[A] at 20:47336426 (ZNFX1)	0.221	0.016	0.012	1.9E-01	4.9E-01	6.9E-01	22264	Same
rs7319045[A] at 13:90822575 (GPC5)	0.397	0.013	0.011	2.1E-01	5.3E-01	8.4E-01	21976	Same
rs3812163[A] at 6:7670759 (BMP6)	0.452	-0.013	0.010	2.2E-01	5.4E-01	8.7E-01	21312	Same
rs5017948[A] at 11:51270794 (OR4A5)	0.188	0.017	0.014	2.2E-01	5.4E-01	8.0E-01	20538	Same
rs12474201[A] at 2:46774789 (SOCS5)	0.349	0.013	0.011	2.2E-01	5.4E-01	5.4E-01	22149	Same
rs6959212[T] at 7:38094851 (STARD3NL)	0.295	-0.012	0.010	2.3E-01	5.4E-01	3.4E-02	27616	Same
rs17081935[T] at 4:57518233 (POLR2B)	0.208	0.013	0.011	2.3E-01	5.4E-01	1.7E-01	28388	Same
rs3129109[T] at 6:29192211 (OR2J3)	0.383	-0.012	0.010	2.3E-01	5.4E-01	9.1E-01	24215	Same
rs12902421[T] at 15:69948457 (MYO9A)	0.027	-0.040	0.034	2.4E-01	5.5E-01	4.9E-01	21434	Same

rs9863706[T] at 3:72520103 (RYBP)	0.210	-0.014	0.012	2.4E-01	5.6E-01	8.8E-01	22230	Same
rs1351164[T] at 2:217980143 (TNS1)	0.218	-0.015	0.013	2.5E-01	5.6E-01	3.7E-01	21431	Opposite
rs7532866[A] at 1:26614131 (LIN28)	0.336	0.011	0.010	2.5E-01	5.6E-01	7.2E-02	27573	Same
rs17511102[A] at 2:37814117 (CDC42EP3)	0.083	-0.022	0.020	2.7E-01	5.9E-01	8.4E-02	21351	Same
rs4965598[T] at 15:98577137 (ADAMTS17)	0.311	0.011	0.010	2.7E-01	5.9E-01	4.2E-01	27536	Opposite
rs16964211[A] at 15:49317787 (CYP19A1)	0.061	0.021	0.019	2.7E-01	5.9E-01	8.1E-01	28409	Opposite
rs9360921[T] at 6:76322362 (SENP6)	0.118	-0.017	0.016	2.8E-01	5.9E-01	7.1E-01	22266	Same
rs2341459[T] at 2:44621706 (C2orf34)	0.302	0.010	0.009	2.9E-01	6.1E-01	9.2E-01	28332	Same
rs11118346[T] at 1:217810342 (LYPLAL1)	0.471	-0.009	0.009	2.9E-01	6.1E-01	3.3E-02	28449	Same
rs17346452[T] at 1:170319910 (DNM3)	0.265	-0.012	0.011	3.1E-01	6.4E-01	8.2E-01	22249	Same
rs7112925[T] at 11:66582736 (RHOD)	0.324	-0.009	0.009	3.1E-01	6.4E-01	9.7E-01	28321	Same
rs7971536[A] at 12:100897919 (CCDC53/GNPTAB)	0.452	-0.010	0.010	3.2E-01	6.4E-01	9.7E-01	22041	Same
rs1570106[T] at 14:67882868 (RAD51L1)	0.201	-0.012	0.013	3.2E-01	6.4E-01	6.2E-01	22255	Same
rs2279008[T] at 19:17144303 (MYO9B)	0.305	0.010	0.010	3.3E-01	6.4E-01	2.1E-01	27329	Same
rs11107116[T] at 12:92502635 (SOCS2)	0.233	-0.010	0.010	3.3E-01	6.4E-01	8.2E-01	28328	Opposite
rs3118905[A] at 13:50003335 (DLEU7)	0.295	-0.011	0.011	3.4E-01	6.4E-01	6.5E-02	22260	Same
rs7027110[A] at 9:108638867 (ZNF462)	0.227	0.010	0.010	3.4E-01	6.4E-01	2.4E-01	28332	Same
rs4282339[A] at 5:168188818 (SLIT3)	0.200	-0.010	0.011	3.4E-01	6.4E-01	5.7E-01	28335	Same
rs654723[A] at 11:128091365 (FLI1)	0.371	-0.009	0.009	3.4E-01	6.4E-01	7.0E-01	28235	Opposite
rs10799445[A] at 1:225978506 (JMJD4)	0.243	0.011	0.012	3.5E-01	6.5E-01	2.0E-02	22262	Same

rs889014[T] at 5:172916720 (BOD1)	0.371	-0.008	0.009	3.5E-01	6.5E-01	8.8E-01	28395	Same
rs7853377[A] at 9:85742025 (C9orf64)	0.221	-0.011	0.012	3.7E-01	6.6E-01	1.3E-01	22201	Same
rs17318596[A] at 19:46628935 (ATP5SL)	0.350	0.008	0.009	3.8E-01	6.6E-01	4.0E-01	28314	Same
rs310405[A] at 6:81857081 (FAM46A)	0.495	-0.008	0.009	3.8E-01	6.6E-01	6.3E-01	28321	Opposite
rs7864648[T] at 9:16358732 (BNC2)	0.339	0.008	0.009	3.8E-01	6.6E-01	5.5E-01	28286	Same
rs7567288[T] at 2:134151294 (NCKAP5)	0.242	-0.009	0.011	3.8E-01	6.6E-01	4.4E-01	28431	Same
rs9844666[A] at 3:137456906 (PCCB)	0.229	0.009	0.010	3.9E-01	6.6E-01	5.9E-01	28336	Opposite
rs4470914[T] at 7:19583047 (TWISTNB)	0.178	-0.012	0.013	3.9E-01	6.6E-01	8.3E-01	22193	Opposite
rs9456307[A] at 6:158849430 (TULP4)	0.061	-0.021	0.024	3.9E-01	6.6E-01	7.0E-01	21425	Same
rs4800452[T] at 18:18981609 (CABLES1)	0.217	0.010	0.011	3.9E-01	6.6E-01	1.0E-01	25046	Same
rs11684404[T] at 2:88705737 (EIF2AK3)	0.370	-0.009	0.011	4.0E-01	6.7E-01	4.9E-01	22194	Same
rs6457620[C] at 6:32771977 (HLA locus)	0.478	-0.008	0.010	4.2E-01	6.9E-01	2.7E-03	22263	Same
rs1046934[A] at 1:182290152 (TSEN15)	0.378	0.007	0.009	4.3E-01	6.9E-01	3.4E-02	28336	Opposite
rs12694997[A] at 2:241911659 (37500)	0.219	-0.008	0.010	4.3E-01	6.9E-01	5.6E-01	28299	Same
rs788867[T] at 4:82369030 (PRKG2)	0.323	0.009	0.012	4.4E-01	7.1E-01	5.5E-01	18023	Opposite
rs7759938[T] at 6:105485647 (LIN28B)	0.324	-0.007	0.010	4.6E-01	7.3E-01	9.5E-01	25036	Same
rs425277[T] at 1:2059032 (PRKCZ)	0.282	-0.007	0.010	4.6E-01	7.3E-01	8.2E-01	27323	Opposite
rs10838801[A] at 11:48054856 (PTPRJ/SLC39A13)	0.312	-0.009	0.012	4.7E-01	7.3E-01	9.1E-01	17620	Same
rs3764419[A] at 17:26188149 (ATAD5/RNF135)	0.395	-0.006	0.009	4.9E-01	7.5E-01	4.9E-01	28322	Same
rs2778031[T] at 9:90025546 (SPIN1)	0.234	-0.008	0.012	4.9E-01	7.5E-01	2.0E-01	22233	Opposite

rs2638953[C] at 12:28425682 (CCDC91)	0.315	0.006	0.009	4.9E-01	7.5E-01	9.3E-01	28433	Same
rs9967417[C] at 18:45213498 (DYM)	0.390	-0.007	0.011	5.0E-01	7.5E-01	4.4E-01	22186	Same
rs11648796[A] at 16:732191 (NARFL)	0.262	-0.009	0.014	5.0E-01	7.5E-01	4.5E-01	17745	Same
rs13177718[T] at 5:108141243 (FER)	0.064	0.012	0.018	5.1E-01	7.5E-01	5.3E-02	27006	Opposite
rs2072153[C] at 17:44745013 (ZNF652)	0.300	-0.008	0.012	5.1E-01	7.5E-01	4.6E-01	18853	Opposite
rs1047014[T] at 6:19949472 (ID4)	0.244	-0.007	0.011	5.2E-01	7.6E-01	4.7E-01	22141	Same
rs17780086[A] at 17:27367395 (LRRC37B)	0.140	0.010	0.015	5.2E-01	7.6E-01	7.3E-01	22264	Same
rs2856321[A] at 12:11747040 (ETV6)	0.367	-0.007	0.010	5.3E-01	7.6E-01	2.4E-01	22205	Same
rs12153391[A] at 5:171136043 (FBXW11)	0.266	-0.006	0.010	5.4E-01	7.7E-01	6.9E-01	27448	Same
rs634552[T] at 11:74959700 (SERPINH1)	0.154	0.009	0.014	5.5E-01	7.8E-01	5.3E-01	22208	Same
rs11144688[A] at 9:77732106 (PCSK5)	0.120	-0.010	0.018	5.5E-01	7.8E-01	3.0E-01	25943	Same
rs7274811[T] at 20:31796842 (ZNF341)	0.241	0.006	0.010	5.8E-01	8.1E-01	2.2E-01	28337	Opposite
rs526896[T] at 5:134384604 (PITX1)	0.270	0.005	0.010	5.9E-01	8.2E-01	4.7E-01	28139	Same
rs720390[A] at 3:187031377 (IGF2BP2)	0.383	-0.005	0.009	6.1E-01	8.3E-01	5.8E-01	27388	Opposite
rs6699417[T] at 1:88896031 (PKN2)	0.364	0.005	0.009	6.1E-01	8.3E-01	8.7E-01	28325	Same
rs8052560[A] at 16:87304743 (CTU2)	0.200	0.008	0.016	6.2E-01	8.3E-01	3.9E-01	19676	Same
rs2110001[C] at 7:150147955 (TMEM176A)	0.338	0.006	0.012	6.2E-01	8.3E-01	6.0E-01	20481	Opposite
rs6714546[A] at 2:33214929 (LTBP1)	0.267	-0.006	0.012	6.3E-01	8.4E-01	3.3E-01	21248	Same
rs17391694[T] at 1:78396214 (GIPC2)	0.129	-0.006	0.013	6.5E-01	8.5E-01	4.5E-01	28328	Opposite
rs822552[C] at 7:148281567 (PDIA4)	0.270	-0.005	0.011	6.5E-01	8.5E-01	6.6E-01	28135	Same

rs17782313[T] at 18:56002077 (MC4R)	0.232	0.005	0.012	6.5E-01	8.5E-01	6.2E-01	23192	Opposite
rs8181166[C] at 9:88306448 (ZCCHC6)	0.472	-0.005	0.010	6.6E-01	8.5E-01	4.4E-01	21348	Opposite
rs6439167[T] at 3:130533446 (C3orf47)	0.208	-0.006	0.013	6.6E-01	8.5E-01	8.3E-01	22265	Same
rs10037512[T] at 5:88390431 (MEF2C)	0.451	0.004	0.010	6.7E-01	8.5E-01	5.8E-01	22260	Same
rs751543[T] at 9:118162163 (PAPPA)	0.284	0.004	0.010	6.8E-01	8.6E-01	2.7E-01	27893	Same
rs9969804[A] at 9:94468941 (IPPK)	0.468	0.004	0.009	6.9E-01	8.6E-01	9.6E-01	24893	Same
rs4605213[C] at 17:46599746 (NME2)	0.336	-0.004	0.011	6.9E-01	8.6E-01	5.2E-01	22103	Opposite
rs2256183[A] at 6:31488508 (MICA)	0.450	-0.004	0.010	6.9E-01	8.6E-01	7.3E-01	22263	Opposite
rs1468758[T] at 9:112846903 (LPAR1)	0.242	0.004	0.010	7.0E-01	8.7E-01	5.0E-01	28134	Opposite
rs7567851[C] at 2:178392966 (PDE11A)	0.077	-0.007	0.019	7.1E-01	8.7E-01	8.7E-01	22254	Opposite
rs4601530[T] at 1:24916698 (CLIC4)	0.275	0.004	0.009	7.1E-01	8.7E-01	4.9E-01	28333	Opposite
rs11599750[T] at 10:101795432 (CPN1)	0.348	0.003	0.009	7.2E-01	8.7E-01	1.7E-01	28320	Opposite
rs1330[T] at 11:17272605 (NUCB2)	0.334	-0.003	0.009	7.3E-01	8.7E-01	9.1E-01	28334	Opposite
rs12470505[T] at 2:219616613 (CCDC108/IHH)	0.093	-0.005	0.015	7.3E-01	8.7E-01	8.0E-01	28446	Opposite
rs7332115[T] at 13:32045548 (PDS5B)	0.395	-0.003	0.009	7.4E-01	8.7E-01	1.7E-01	28328	Same
rs4640244[A] at 17:21224816 (KCNJ12)	0.402	0.003	0.009	7.6E-01	8.9E-01	6.6E-01	27224	Same
rs572169[T] at 3:173648421 (GHSR)	0.321	-0.003	0.009	7.7E-01	9.0E-01	2.8E-01	28333	Opposite
rs10770705[A] at 12:20748734 (SLCO1C1)	0.323	-0.003	0.009	7.7E-01	9.0E-01	8.3E-03	28333	Opposite
rs10874746[T] at 1:93096559 (RPL5)	0.373	0.002	0.009	7.9E-01	9.2E-01	3.8E-01	28435	Opposite
rs4665736[T] at 2:25041103 (DNAJC27)	0.476	0.003	0.010	8.0E-01	9.2E-01	3.4E-01	22194	Same

rs2665838[C] at 17:59320197 (CSH1/GH1)	0.261	-0.003	0.012	8.2E-01	9.2E-01	5.3E-01	22150	Same
rs2145998[A] at 10:80791702 (PPIF)	0.496	0.002	0.010	8.3E-01	9.2E-01	2.4E-01	22244	Opposite
rs2834442[A] at 21:34612656 (KCNE2)	0.345	-0.002	0.010	8.3E-01	9.2E-01	5.2E-01	22258	Opposite
rs9835332[C] at 3:56642722 (C3orf63)	0.470	-0.002	0.010	8.3E-01	9.2E-01	8.7E-01	22262	Same
rs11958779[A] at 5:55037656 (SLC38A9)	0.311	0.002	0.012	8.4E-01	9.2E-01	7.7E-01	18021	Opposite
rs543650[T] at 6:152152636 (ESR1)	0.428	0.002	0.009	8.4E-01	9.2E-01	2.0E-01	28295	Opposite
rs12982744[C] at 19:2128193 (DOT1L)	0.371	0.002	0.011	8.5E-01	9.2E-01	1.9E-01	21144	Opposite
rs227724[A] at 17:52133816 (NOG)	0.355	-0.002	0.011	8.5E-01	9.2E-01	8.5E-01	22214	Same
rs12680655[C] at 8:135706519 (ZFAT)	0.424	0.002	0.010	8.6E-01	9.2E-01	6.1E-01	22256	Same
rs26868[A] at 16:2189377 (CASKIN1)	0.456	0.002	0.011	8.6E-01	9.2E-01	5.9E-01	20302	Same
rs2066807[C] at 12:55026949 (STAT2)	0.059	0.003	0.019	8.6E-01	9.2E-01	3.2E-01	27132	Opposite
rs955748[A] at 4:184452669 (WWC2)	0.237	-0.002	0.010	8.7E-01	9.2E-01	2.7E-01	28326	Same
rs3782089[T] at 11:65093395 (SSSCA1)	0.069	-0.003	0.018	8.7E-01	9.2E-01	2.1E-02	28428	Same
rs7909670[T] at 10:12958770 (CCDC3)	0.451	-0.001	0.010	8.9E-01	9.3E-01	5.7E-01	23013	Same
rs13088462[T] at 3:51046753 (DOCK3)	0.060	-0.002	0.019	9.0E-01	9.4E-01	8.1E-01	26998	Same
rs2580816[T] at 2:232506210 (NPPC)	0.184	-0.002	0.013	9.0E-01	9.4E-01	4.9E-01	22136	Same
rs274546[A] at 5:131727766 (SLC22A5)	0.429	-0.001	0.009	9.1E-01	9.4E-01	1.6E-01	28453	Same
rs2079795[T] at 17:56851431 (TBX2)	0.306	0.001	0.010	9.2E-01	9.5E-01	9.8E-01	28386	Same
rs1741344[T] at 20:4049800 (SMOX)	0.356	-0.001	0.009	9.3E-01	9.5E-01	7.6E-01	28320	Same
rs7697556[T] at 4:73734177 (ADAMTS3)	0.484	0.001	0.009	9.5E-01	9.6E-01	6.4E-01	28072	Same

rs10152591[A] at 15:67835211 (TLE3)	0.087	-0.001	0.016	9.6E-01	9.7E-01	4.9E-01	28337	Opposite
rs1738475[C] at 1:23409478 (HTR1D)	0.406	0.000	0.010	9.7E-01	9.7E-01	6.9E-01	22263	Opposite
rs17806888[T] at 3:67499012 (SUCLG2)	0.107	0.000	0.016	1.0E+00	1.0E+00	7.9E-01	28446	Opposite

Single nucleotide polymorphisms (SNPs) markers are identified according to their standard rs numbers (NCBI build 36). The total sample includes data of 22 independent datasets (N = 28,459). *: One SNP (rs724577) at this known adult height locus was genome-wide significant in our discovery analysis. MAF, minor allele frequency; S.E., standard error. *ß* reflects differences in standardized birth length per effect allele. *P* values are obtained from linear regression of each SNP against standardized birth length adjusted for sex and gestational age (**Bold**: *P*_False-Discovery-Rate < 0.05). *HetP* values reflect heterogeneity across discovery studies with the use of Cochran's Q tests. We included both GWA and metabochip cohorts in our discovery analysis, this explains the differences in numbers (n). GIANT, is the allele effect in the GIANT paper.

Table S8. Summary statistics of the known adult height loci and their association with infant length (N = 28,238).

**Marker[effect allele]**	**MAF**	***ß***	**S.E.**	***P***	***P*_FDR**	***HetP***	**n**	**GIANT**
rs11205277[A] at 1:148159496 (SF3B4)*	0.375	-0.074	0.010	8.8E-15	**1.6E-12**	9.2E-01	28232	Same
rs6449353[T] at 4:17642586 (LCORL)*	0.142	0.098	0.013	1.1E-13	**1.0E-11**	1.2E-01	28232	Same
rs143384[A] at 20:33489170 (GDF5)*	0.441	-0.058	0.009	2.9E-10	**1.7E-08**	1.0E+00	28232	Same
rs1490384[T] at 6:126892853 (C6orf173)*	0.496	0.051	0.009	2.8E-09	**1.1E-07**	8.2E-01	28225	Same
rs7689420[T] at 4:145787802 (HHIP)*	0.176	-0.068	0.011	3.1E-09	**1.1E-07**	4.0E-01	28229	Same
rs12534093[A] at 7:23469499 (IGF2BP3)	0.221	-0.064	0.012	8.2E-08	**2.1E-06**	1.6E-02	23080	Same
rs237743[A] at 20:47336426 (ZNFX1)*	0.212	0.062	0.012	8.9E-08	**2.1E-06**	1.2E-01	23079	Same
rs9428104[A] at 1:118657110 (SPAG17)*	0.245	-0.061	0.011	9.3E-08	**2.1E-06**	5.3E-01	23080	Same
rs1351394[T] at 12:64638093 (HMGA2)*	0.496	0.046	0.009	1.4E-07	**2.8E-06**	3.7E-01	27440	Same
rs724016[A] at 3:142588260 (ZBTB38)	0.453	-0.044	0.009	3.7E-07	**6.7E-06**	7.0E-01	28230	Same
rs6470764[T] at 8:130794847 (GSDMC)	0.203	-0.054	0.011	1.1E-06	**1.8E-05**	5.8E-01	28227	Same
rs6473015[A] at 8:78341040 (PEX2)	0.263	-0.045	0.010	4.2E-06	**6.2E-05**	6.7E-01	28228	Same
rs1325598[A] at 1:175058872 (PAPPA2)	0.439	-0.044	0.010	9.7E-06	**1.3E-04**	5.0E-01	22294	Same
rs11259936[A] at 15:82371586 (ADAMTSL3)	0.474	-0.038	0.009	1.4E-05	**1.6E-04**	8.4E-01	27440	Same
rs17081935[T] at 4:57518233 (POLR2B)	0.210	0.046	0.011	1.4E-05	**1.6E-04**	4.3E-01	28223	Same
rs11118346[T] at 1:217810342 (LYPLAL1)	0.472	-0.037	0.009	1.6E-05	**1.8E-04**	4.4E-01	28229	Same
rs6457620[C] at 6:32771977 (HLA locus)*	0.473	-0.041	0.010	1.7E-05	**1.8E-04**	8.5E-01	23077	Same

rs2093210[T] at 14:60027032 (SIX6)	0.418	-0.045	0.010	1.8E-05	**1.8E-04**	3.2E-01	23080	Same
rs11830103[A] at 12:122389499 (SBNO1)	0.211	-0.056	0.013	2.0E-05	**1.9E-04**	8.5E-01	19910	Same
rs473902[T] at 9:97296056 (PTCH1)*	0.082	0.076	0.019	3.7E-05	**3.4E-04**	2.1E-01	28231	Same
rs806794[A] at 6:26308656 (Histone cluster)	0.345	0.039	0.009	4.3E-05	**3.7E-04**	4.8E-01	27439	Same
rs7759938[T] at 6:105485647 (LIN28B)	0.320	-0.039	0.010	5.8E-05	**4.7E-04**	7.6E-01	25835	Same
rs2145272[A] at 20:6574218 (BMP2)	0.333	-0.035	0.009	1.3E-04	**9.9E-04**	6.4E-01	27448	Same
rs3791675[T] at 2:55964813 (EFEMP1)	0.240	-0.039	0.010	1.3E-04	**9.9E-04**	1.4E-01	28230	Same
rs2154319[T] at 1:41518357 (SCMH1)	0.236	-0.046	0.012	1.6E-04	**1.1E-03**	3.1E-01	22296	Same
rs1708299[A] at 7:28156471 (JAZF1)	0.302	0.039	0.011	2.4E-04	**1.6E-03**	4.5E-01	23320	Same
rs16942341[T] at 15:87189909 (ACAN)*	0.026	-0.112	0.031	3.0E-04	**2.0E-03**	7.3E-01	27075	Same
rs13177718[T] at 5:108141243 (FER)	0.063	-0.065	0.018	3.2E-04	**2.1E-03**	1.5E-01	28230	Same
rs42235[T] at 7:92086012 (CDK6)	0.295	0.036	0.010	5.8E-04	**3.5E-03**	2.8E-01	24272	Same
rs2871865[C] at 15:97012419 (IGF1R)	0.116	0.059	0.017	5.8E-04	**3.5E-03**	4.8E-01	22291	Same
rs7926971[A] at 11:12654616 (TEAD1)	0.454	-0.032	0.010	9.6E-04	**5.6E-03**	3.9E-01	23077	Same
rs9456307[A] at 6:158849430 (TULP4)	0.063	-0.074	0.023	1.1E-03	**6.4E-03**	6.3E-02	22296	Same
rs7178424[T] at 15:60167551 (C2CD4A)	0.474	-0.029	0.009	1.3E-03	**6.9E-03**	7.2E-01	27442	Same
rs5742915[T] at 15:72123686 (PML)	0.432	-0.029	0.009	1.3E-03	**7.1E-03**	3.4E-01	27431	Same
rs2247341[A] at 4:1671115 (SLBP)	0.381	0.028	0.009	1.4E-03	**7.2E-03**	3.0E-01	28231	Same
rs1257763[A] at 9:95933766 (PTPDC1)	0.041	0.083	0.026	1.5E-03	**7.2E-03**	1.6E-01	25775	Same
rs572169[T] at 3:173648421 (GHSR)	0.316	0.029	0.009	1.5E-03	**7.2E-03**	1.4E-01	28232	Same

rs3812163[A] at 6:7670759 (BMP6)	0.449	-0.031	0.010	1.6E-03	**7.4E-03**	6.9E-01	23079	Same
rs7460090[T] at 8:57356717 (SDR16C5)	0.130	0.046	0.015	1.7E-03	**8.0E-03**	9.6E-01	23079	Same
rs4821083[T] at 22:31386341 (SYN3)	0.152	0.037	0.012	1.9E-03	**8.6E-03**	5.7E-01	28226	Same
rs1046943[A] at 6:109890634 (ZBTB24)	0.438	0.030	0.010	2.1E-03	**9.2E-03**	9.2E-01	23077	Same
rs2638953[C] at 12:28425682 (CCDC91)	0.318	0.028	0.009	2.3E-03	**9.9E-03**	5.3E-01	28218	Same
rs955748[A] at 4:184452669 (WWC2)	0.243	-0.030	0.010	2.7E-03	**1.1E-02**	3.9E-02	28224	Same
rs10748128[T] at 12:68113925 (FRS2)	0.351	0.028	0.010	3.9E-03	**1.6E-02**	3.5E-01	25840	Same
rs3110496[A] at 17:24941897 (ANKRD13B)	0.356	-0.026	0.009	4.1E-03	**1.7E-02**	8.2E-01	28225	Same
rs1351164[T] at 2:217980143 (TNS1)	0.218	0.034	0.012	5.2E-03	**2.0E-02**	8.2E-02	22296	Same
rs9863706[T] at 3:72520103 (RYBP)	0.211	-0.033	0.012	5.5E-03	**2.1E-02**	1.9E-01	23075	Same
rs7763064[A] at 6:142838982 (GPR126)	0.289	-0.026	0.010	5.9E-03	**2.2E-02**	6.4E-03	28230	Same
rs9969804[A] at 9:94468941 (IPPK)	0.463	0.024	0.009	7.4E-03	**2.7E-02**	3.6E-01	25816	Same
rs11107116[T] at 12:92502635 (SOCS2)	0.230	-0.027	0.010	7.6E-03	**2.7E-02**	7.1E-01	28228	Opposite
rs798489[T] at 7:2768329 (GNA12)	0.302	-0.025	0.009	7.9E-03	**2.8E-02**	6.8E-01	28227	Same
rs1330[T] at 11:17272605 (NUCB2)	0.336	0.023	0.009	1.1E-02	**3.8E-02**	4.9E-01	28230	Same
rs1043515[A] at 17:34175722 (PIP4K2B)	0.461	-0.022	0.009	1.1E-02	**3.8E-02**	9.9E-01	28227	Same
rs1950500[T] at 14:23900690 (NFATC4)	0.289	0.024	0.009	1.1E-02	**3.8E-02**	5.4E-01	28231	Same
rs7027110[A] at 9:108638867 (ZNF462)	0.227	0.026	0.010	1.2E-02	**3.9E-02**	1.7E-01	28227	Same
rs7849585[T] at 9:138251691 (QSOX2)	0.341	0.026	0.010	1.3E-02	**4.0E-02**	3.6E-01	23074	Same
rs11144688[A] at 9:77732106 (PCSK5)	0.120	-0.043	0.017	1.3E-02	**4.2E-02**	6.9E-01	26324	Same

rs2256183[A] at 6:31488508 (MICA)	0.442	0.024	0.010	1.4E-02	**4.4E-02**	6.8E-01	23078	Same
rs2780226[T] at 6:34307070 (HMGA1)	0.078	-0.043	0.018	1.6E-02	**4.8E-02**	2.1E-01	25845	Same
rs4800452[T] at 18:18981609 (CABLES1)	0.217	0.026	0.011	1.7E-02	5.2E-02	1.4E-02	25847	Same
rs10010325[A] at 4:106325802 (TET2)	0.463	0.021	0.009	1.8E-02	5.2E-02	6.7E-02	28214	Same
rs7332115[T] at 13:32045548 (PDS5B)	0.395	-0.021	0.009	1.9E-02	5.6E-02	7.6E-01	28226	Same
rs4072910[C] at 19:8550031 (ADAMTS10)	0.453	-0.026	0.011	2.0E-02	5.7E-02	7.6E-01	22065	Same
rs891088[A] at 19:7135762 (INSR)	0.278	-0.022	0.010	2.0E-02	5.7E-02	8.3E-01	28224	Same
rs2778031[T] at 9:90025546 (SPIN1)	0.239	0.026	0.012	2.7E-02	7.3E-02	8.1E-01	23079	Same
rs6439167[T] at 3:130533446 (C3orf47)	0.202	-0.027	0.012	2.7E-02	7.3E-02	3.4E-01	23080	Same
rs10863936[A] at 1:210304421 (DTL)	0.475	-0.019	0.009	2.7E-02	7.3E-02	3.6E-01	28220	Same
rs16964211[A] at 15:49317787 (CYP19A1)	0.064	-0.042	0.019	2.8E-02	7.4E-02	2.6E-02	27401	Same
rs12694997[A] at 2:241911659 (37500)	0.221	-0.022	0.010	2.9E-02	7.4E-02	4.2E-01	28202	Same
rs274546[A] at 5:131727766 (SLC22A5)	0.430	-0.019	0.009	2.9E-02	7.4E-02	3.0E-01	28229	Same
rs17391694[T] at 1:78396214 (GIPC2)	0.127	0.028	0.013	3.1E-02	8.0E-02	6.4E-01	28232	Same
rs4470914[T] at 7:19583047 (TWISTNB)	0.181	0.027	0.013	3.5E-02	8.7E-02	4.5E-01	23080	Same
rs10874746[T] at 1:93096559 (RPL5)	0.372	-0.018	0.009	3.8E-02	9.1E-02	4.1E-01	28218	Same
rs11648796[A] at 16:732191 (NARFL)	0.272	-0.027	0.013	3.8E-02	9.1E-02	3.4E-01	19904	Same
rs26868[A] at 16:2189377 (CASKIN1)	0.445	0.022	0.011	3.8E-02	9.1E-02	4.8E-01	20489	Same
rs7319045[A] at 13:90822575 (GPC5)	0.400	0.021	0.010	4.1E-02	9.6E-02	3.0E-01	23080	Same
rs12902421[T] at 15:69948457 (MYO9A)	0.029	-0.066	0.032	4.1E-02	9.7E-02	4.4E-01	19833	Same

rs7971536[A] at 12:100897919 (CCDC53/GNPTAB)	0.448	-0.020	0.010	4.3E-02	1.0E-01	7.6E-01	23080	Same
rs526896[T] at 5:134384604 (PITX1)	0.270	0.020	0.010	4.5E-02	1.0E-01	1.2E-01	28231	Same
rs822552[C] at 7:148281567 (PDIA4)	0.273	-0.021	0.011	4.9E-02	1.1E-01	2.4E-01	28232	Same
rs11958779[A] at 5:55037656 (SLC38A9)	0.310	-0.023	0.011	4.9E-02	1.1E-01	4.5E-01	19909	Same
rs7909670[T] at 10:12958770 (CCDC3)	0.451	-0.018	0.010	5.5E-02	1.2E-01	8.9E-01	24058	Same
rs12153391[A] at 5:171136043 (FBXW11)	0.265	-0.020	0.010	5.6E-02	1.2E-01	5.5E-01	27444	Same
rs7274811[T] at 20:31796842 (ZNF341)	0.239	-0.019	0.010	5.7E-02	1.2E-01	3.4E-02	28231	Same
rs2665838[C] at 17:59320197 (CSH1/GH1)	0.267	-0.021	0.011	5.8E-02	1.2E-01	6.6E-01	23079	Same
rs7567288[T] at 2:134151294 (NCKAP5)	0.240	-0.019	0.010	6.3E-02	1.3E-01	4.1E-01	28233	Same
rs6879260[T] at 5:179663620 (GFPT2)	0.399	-0.016	0.009	6.6E-02	1.3E-01	6.0E-01	28223	Same
rs17780086[A] at 17:27367395 (LRRC37B)	0.136	0.026	0.014	6.6E-02	1.3E-01	9.3E-01	23079	Same
rs961764[C] at 6:117628849 (VGLL2)	0.414	-0.018	0.010	6.6E-02	1.3E-01	7.4E-01	23077	Same
rs17806888[T] at 3:67499012 (SUCLG2)	0.106	0.028	0.015	6.8E-02	1.3E-01	8.8E-01	28230	Same
rs227724[A] at 17:52133816 (NOG)	0.354	-0.019	0.010	6.8E-02	1.3E-01	8.1E-01	23079	Same
rs6569648[T] at 6:130390812 (L3MBTL3)	0.221	-0.018	0.010	7.0E-02	1.4E-01	6.9E-01	28226	Same
rs12474201[A] at 2:46774789 (SOCS5)	0.342	0.019	0.010	7.0E-02	1.4E-01	8.3E-01	23079	Same
rs7155279[T] at 14:91555634 (TRIP11)	0.365	-0.018	0.010	7.4E-02	1.4E-01	7.8E-01	23078	Same
rs17318596[A] at 19:46628935 (ATP5SL)	0.348	0.016	0.009	7.8E-02	1.5E-01	1.3E-01	28214	Same
rs2580816[T] at 2:232506210 (NPPC)	0.191	-0.022	0.013	8.1E-02	1.5E-01	6.0E-01	23082	Same
rs1047014[T] at 6:19949472 (ID4)	0.241	-0.019	0.011	8.8E-02	1.6E-01	9.6E-01	23079	Same

rs17511102[A] at 2:37814117 (CDC42EP3)	0.084	-0.032	0.019	8.9E-02	1.6E-01	2.2E-01	22296	Same
rs4601530[T] at 1:24916698 (CLIC4)	0.276	-0.016	0.010	9.9E-02	1.8E-01	4.3E-01	28223	Same
rs862034[A] at 14:74060499 (LTBP2)	0.351	-0.017	0.010	1.0E-01	1.9E-01	2.7E-01	22294	Same
rs12470505[T] at 2:219616613 (CCDC108/IHH)	0.100	0.024	0.015	1.1E-01	1.9E-01	6.2E-01	28232	Same
rs8181166[C] at 9:88306448 (ZCCHC6)	0.470	0.016	0.010	1.1E-01	1.9E-01	5.1E-01	22294	Same
rs1468758[T] at 9:112846903 (LPAR1)	0.245	-0.016	0.010	1.1E-01	2.0E-01	6.2E-01	28232	Same
rs2066807[C] at 12:55026949 (STAT2)	0.059	-0.029	0.018	1.2E-01	2.0E-01	4.5E-01	28230	Same
rs7697556[T] at 4:73734177 (ADAMTS3)	0.484	0.013	0.009	1.2E-01	2.1E-01	8.9E-01	27859	Same
rs7532866[A] at 1:26614131 (LIN28)	0.330	0.014	0.009	1.3E-01	2.2E-01	5.8E-01	27448	Same
rs10770705[A] at 12:20748734 (SLCO1C1)	0.320	0.014	0.009	1.3E-01	2.2E-01	4.0E-01	28232	Same
rs3129109[T] at 6:29192211 (OR2J3)	0.384	-0.014	0.010	1.4E-01	2.4E-01	7.7E-01	25062	Same
rs1659127[A] at 16:14295806 (MKL2)	0.331	0.014	0.010	1.5E-01	2.4E-01	1.5E-01	28227	Same
rs10799445[A] at 1:225978506 (JMJD4)	0.243	0.016	0.011	1.5E-01	2.5E-01	3.3E-02	23078	Same
rs2079795[T] at 17:56851431 (TBX2)	0.314	0.013	0.009	1.6E-01	2.5E-01	8.5E-01	28230	Same
rs10838801[A] at 11:48054856 (PTPRJ/SLC39A13)	0.313	-0.016	0.012	1.7E-01	2.7E-01	9.1E-01	19521	Same
rs1046934[A] at 1:182290152 (TSEN15)	0.375	-0.012	0.009	1.8E-01	2.9E-01	1.6E-02	28231	Same
rs7567851[C] at 2:178392966 (PDE11A)	0.083	0.024	0.018	1.9E-01	3.0E-01	6.6E-01	23052	Same
rs2597513[T] at 3:13530836 (HDAC11)	0.101	-0.019	0.015	1.9E-01	3.0E-01	2.3E-01	28232	Same
rs3118905[A] at 13:50003335 (DLEU7)	0.288	-0.014	0.011	2.0E-01	3.1E-01	2.9E-01	23078	Same
rs2237886[T] at 11:2767307 (KCNQ1)	0.095	0.019	0.015	2.0E-01	3.1E-01	2.7E-01	28229	Same

rs4986172[T] at 17:40571807 (ACBD4)	0.362	-0.011	0.009	2.1E-01	3.2E-01	4.9E-02	28221	Same
rs17346452[T] at 1:170319910 (DNM3)	0.262	-0.014	0.011	2.1E-01	3.2E-01	6.4E-02	23079	Same
rs1582931[A] at 5:122685098 (CEP120)	0.490	0.013	0.010	2.1E-01	3.2E-01	2.5E-01	23073	Opposite
rs889014[T] at 5:172916720 (BOD1)	0.373	-0.011	0.009	2.2E-01	3.3E-01	7.1E-01	28227	Same
rs4605213[C] at 17:46599746 (NME2)	0.335	0.013	0.011	2.2E-01	3.3E-01	9.1E-02	23080	Same
rs751543[T] at 9:118162163 (PAPPA)	0.283	0.012	0.010	2.2E-01	3.3E-01	7.8E-01	28231	Same
rs6959212[T] at 7:38094851 (STARD3NL)	0.300	-0.011	0.010	2.4E-01	3.5E-01	4.8E-01	27439	Same
rs3764419[A] at 17:26188149 (ATAD5/RNF135)	0.391	-0.010	0.009	2.4E-01	3.5E-01	8.5E-01	28213	Same
rs6699417[T] at 1:88896031 (PKN2)	0.363	0.010	0.009	2.5E-01	3.5E-01	4.5E-01	28213	Same
rs7466269[A] at 9:132453905 (FUBP3)	0.388	0.010	0.009	2.6E-01	3.7E-01	6.0E-01	28225	Same
rs10037512[T] at 5:88390431 (MEF2C)	0.457	-0.011	0.010	2.6E-01	3.7E-01	5.0E-01	23079	Opposite
rs7853377[A] at 9:85742025 (C9orf64)	0.226	-0.012	0.012	3.0E-01	4.1E-01	4.6E-01	23079	Same
rs2336725[T] at 3:53093779 (RTF1)	0.432	-0.009	0.009	3.0E-01	4.1E-01	5.2E-01	28229	Same
rs2110001[C] at 7:150147955 (TMEM176A)	0.332	-0.011	0.011	3.1E-01	4.3E-01	6.0E-01	21908	Same
rs9472414[A] at 6:45054484 (SUPT3H/RUNX2)	0.215	-0.012	0.012	3.2E-01	4.3E-01	6.6E-01	23078	Same
rs1013209[T] at 8:24172249 (ADAM28)	0.253	-0.011	0.011	3.2E-01	4.3E-01	9.0E-01	23079	Same
rs4965598[T] at 15:98577137 (ADAMTS17)	0.310	-0.009	0.010	3.3E-01	4.4E-01	3.7E-02	26580	Same
rs12982744[C] at 19:2128193 (DOT1L)	0.379	-0.010	0.010	3.3E-01	4.4E-01	6.5E-01	23078	Same
rs9360921[T] at 6:76322362 (SENP6)	0.116	-0.014	0.015	3.4E-01	4.5E-01	1.6E-01	23080	Same
rs4282339[A] at 5:168188818 (SLIT3)	0.199	-0.010	0.011	3.6E-01	4.7E-01	7.2E-01	28230	Same

rs2284746[C] at 1:17179262 (MFAP2)	0.499	-0.009	0.010	3.7E-01	4.8E-01	2.2E-01	23079	Same
rs2072153[C] at 17:44745013 (ZNF652)	0.300	-0.010	0.011	3.8E-01	4.9E-01	7.6E-01	20693	Opposite
rs1738475[C] at 1:23409478 (HTR1D)	0.402	0.008	0.010	4.0E-01	5.2E-01	2.3E-01	23077	Same
rs13088462[T] at 3:51046753 (DOCK3)	0.059	-0.015	0.019	4.3E-01	5.4E-01	9.5E-02	26557	Same
rs10152591[A] at 15:67835211 (TLE3)	0.087	0.013	0.016	4.3E-01	5.4E-01	1.6E-01	27443	Same
rs6684205[A] at 1:216676325 (TGFB2)	0.299	0.008	0.010	4.3E-01	5.5E-01	9.5E-01	24099	Opposite
rs9967417[C] at 18:45213498 (DYM)	0.394	-0.008	0.010	4.6E-01	5.7E-01	7.9E-01	23079	Same
rs8052560[A] at 16:87304743 (CTU2)	0.198	0.010	0.014	4.6E-01	5.7E-01	5.3E-01	22297	Same
rs12680655[C] at 8:135706519 (ZFAT)	0.419	0.007	0.010	4.8E-01	5.9E-01	1.1E-01	23077	Same
rs1570106[T] at 14:67882868 (RAD51L1)	0.200	-0.009	0.012	4.8E-01	5.9E-01	4.9E-01	23080	Same
rs11867479[T] at 17:65601802 (KCNJ16)	0.325	0.006	0.009	5.1E-01	6.2E-01	4.3E-01	28233	Same
rs5017948[A] at 11:51270794 (OR4A5)	0.190	0.009	0.013	5.2E-01	6.2E-01	9.3E-01	22692	Same
rs2341459[T] at 2:44621706 (C2orf34)	0.299	0.006	0.009	5.2E-01	6.2E-01	4.1E-01	28229	Same
rs2834442[A] at 21:34612656 (KCNE2)	0.345	0.007	0.010	5.2E-01	6.2E-01	9.4E-01	23078	Same
rs1173727[T] at 5:32866278 (NPR3)	0.398	0.006	0.010	5.3E-01	6.3E-01	9.3E-01	23078	Same
rs17782313[T] at 18:56002077 (MC4R)	0.230	0.007	0.011	5.3E-01	6.3E-01	1.3E-01	24107	Opposite
rs310405[A] at 6:81857081 (FAM46A)	0.495	0.005	0.009	5.4E-01	6.4E-01	4.9E-01	28220	Same
rs11599750[T] at 10:101795432 (CPN1)	0.351	0.005	0.009	5.5E-01	6.4E-01	1.3E-01	28221	Opposite
rs425277[T] at 1:2059032 (PRKCZ)	0.282	-0.006	0.010	5.5E-01	6.4E-01	5.8E-02	28225	Opposite
rs788867[T] at 4:82369030 (PRKG2)	0.326	-0.007	0.011	5.6E-01	6.4E-01	3.6E-01	19910	Same

rs422421[T] at 5:176449932 (FGFR4/NSD1)	0.200	0.006	0.011	5.7E-01	6.5E-01	5.9E-01	28226	Opposite
rs720390[A] at 3:187031377 (IGF2BP2)	0.384	-0.005	0.009	5.8E-01	6.6E-01	4.0E-01	27425	Opposite
rs2279008[T] at 19:17144303 (MYO9B)	0.302	0.005	0.010	5.8E-01	6.6E-01	3.5E-01	27202	Same
rs4665736[T] at 2:25041103 (DNAJC27)	0.467	-0.005	0.010	5.9E-01	6.6E-01	8.8E-01	23080	Opposite
rs7507204[C] at 19:3379834 (NFIC)	0.244	0.007	0.013	6.0E-01	6.7E-01	3.4E-01	20695	Same
rs9835332[C] at 3:56642722 (C3orf63)	0.476	-0.005	0.010	6.4E-01	7.0E-01	1.0E+00	23078	Same
rs7864648[T] at 9:16358732 (BNC2)	0.340	0.004	0.009	6.5E-01	7.1E-01	1.2E-01	28176	Same
rs7112925[T] at 11:66582736 (RHOD)	0.329	0.004	0.009	6.5E-01	7.1E-01	3.7E-01	28177	Opposite
rs6457821[A] at 6:35510783 (PPARD/FANCE)	0.022	-0.018	0.040	6.5E-01	7.1E-01	4.7E-01	17626	Same
rs494459[T] at 11:118079885 (TREH)	0.408	0.003	0.009	6.9E-01	7.4E-01	8.4E-02	28229	Same
rs4640244[A] at 17:21224816 (KCNJ12)	0.394	0.004	0.009	7.0E-01	7.5E-01	8.1E-01	28231	Same
rs9844666[A] at 3:137456906 (PCCB)	0.226	-0.004	0.010	7.0E-01	7.5E-01	6.3E-01	28231	Same
rs2856321[A] at 12:11747040 (ETV6)	0.370	-0.004	0.010	7.1E-01	7.5E-01	6.0E-01	23077	Same
rs2145998[A] at 10:80791702 (PPIF)	0.496	-0.002	0.010	8.1E-01	8.5E-01	9.7E-02	23078	Same
rs1814175[T] at 11:49515748 (FOLH1)	0.378	0.002	0.009	8.2E-01	8.6E-01	1.4E-01	27400	Same
rs1741344[T] at 20:4049800 (SMOX)	0.359	0.002	0.009	8.5E-01	8.8E-01	8.0E-02	28232	Opposite
rs2629046[T] at 2:224755988 (SERPINE2)	0.451	-0.002	0.010	8.5E-01	8.8E-01	3.9E-01	23076	Opposite
rs11684404[T] at 2:88705737 (EIF2AK3)	0.361	-0.002	0.010	8.6E-01	8.9E-01	9.8E-01	23079	Same
rs3782089[T] at 11:65093395 (SSSCA1)	0.068	0.003	0.017	8.7E-01	8.9E-01	5.7E-01	28233	Opposite
rs654723[A] at 11:128091365 (FLI1)	0.375	-0.001	0.009	9.0E-01	9.1E-01	5.4E-01	28187	Opposite

rs634552[T] at 11:74959700 (SERPINH1)	0.159	0.002	0.014	9.0E-01	9.1E-01	3.3E-01	23080	Same
rs6714546[A] at 2:33214929 (LTBP1)	0.271	0.001	0.011	9.3E-01	9.3E-01	5.5E-01	22297	Opposite
rs543650[T] at 6:152152636 (ESR1)	0.424	0.000	0.009	9.8E-01	9.8E-01	1.8E-01	27178	Opposite

Single nucleotide polymorphisms (SNPs) markers are identified according to their standard rs numbers (NCBI build 36). The total sample includes data of 19 independent datasets (N = 28,238). *: At least one SNP at this locus was genomewide significant. MAF, minor allele frequency; S.E., standard error. *ß* reflects differences in standardized infant length per effect allele. *P* values are obtained from linear regression of each SNP against standardized infant length adjusted for sex and age (**Bold**: *P*_False-Discovery-Rate < 0.05; **Red**: *P*_False-Discovery-Rate < 0.05, but effect in wrong direction). *HetP* values reflect heterogeneity across discovery studies with the use of Cochran's Q tests. We included both GWA and metabochip cohorts in our discovery analysis, this explains the differences in numbers (n). GIANT, is the allele effect in the GIANT paper.

REFERENCES

1 Manavathi, B., Lo, D., Bugide, S., Dey, O., Imren, S., Weiss, M.J. and Humphries, R.K. (2012) Functional regulation of pre-B-cell leukemia homeobox interacting protein 1 (PBXIP1/HPIP) in erythroid differentiation. *J. Biol. Chem.*, **287**, 5600-5614.

2 Stone, D.L., Tayebi, N., Orvisky, E., Stubblefield, B., Madike, V. and Sidransky, E. (2000) Glucocerebrosidase gene mutations in patients with type 2 Gaucher disease. *Hum. Mutat.*, **15**, 181-188.

3 Sidransky, E., Nalls, M.A., Aasly, J.O., Aharon-Peretz, J., Annesi, G., Barbosa, E.R., Bar-Shira, A., Berg, D., Bras, J., Brice, A. *et al.* (2009) Multicenter analysis of glucocerebrosidase mutations in Parkinson's disease. *N. Engl. J. Med.*, **361**, 1651-1661.

4 Chahine, L.M., Qiang, J., Ashbridge, E., Minger, J., Yearout, D., Horn, S., Colcher, A., Hurtig, H.I., Lee, V.M., Van Deerlin, V.M. *et al.* (2013) Clinical and biochemical differences in patients having Parkinson disease with vs without GBA mutations. *JAMA Neurol.*, **70**, 852-858.

5 Nalls, M.A., Duran, R., Lopez, G., Kurzawa-Akanbi, M., McKeith, I.G., Chinnery, P.F., Morris, C.M., Theuns, J., Crosiers, D., Cras, P. *et al.* (2013) A multicenter study of glucocerebrosidase mutations in dementia with Lewy bodies. *JAMA Neurol.*, **70**, 727-735.

6 Inoue, D., Reid, M., Lum, L., Kratzschmar, J., Weskamp, G., Myung, Y.M., Baron, R. and Blobel, C.P. (1998) Cloning and initial characterization of mouse meltrin beta and analysis of the expression of four metalloprotease-disintegrins in bone cells. *J. Biol. Chem.*, **273**, 4180-4187.

7 Marzia, M., Guaiquil, V., Horne, W.C., Blobel, C.P., Baron, R. and Chiusaroli, R. (2011) Lack of ADAM15 in mice is associated with increased osteoblast function and bone mass. *Biol. Chem.*, **392**, 877-885.
